# Effects of insecticides and repellents on the spread of ‘*Candidatus* Phytoplasma solani’ under laboratory and field conditions

**DOI:** 10.1007/s41348-023-00768-y

**Published:** 2023-07-04

**Authors:** Monika Riedle-Bauer, Günter Brader

**Affiliations:** 1 Federal College and Research Institute for Viticulture and Pomology Klosterneuburg, Wienerstraße 74, 3400 Klosterneuburg, Austria; 2 AIT Austrian Institute of Technology GmbH, Konrad-Lorenz-Straße 24, 3430 Tulln, Austria

**Keywords:** Stolbur, Potato, Transmission experiment, Rubbery tubers, Management

## Abstract

Recent outbreaks of ‘*Candidatus* Phytoplasma solani’ resulted in severe losses in potatoes, vegetable crops and grapevines in certain regions of Austria and constituted a major challenge for seed potato production. Therefore, the effects of various insecticides and insect deterrents on pathogen spread were studied both in laboratory and field experiments from 2018 to 2021. In laboratory transmission experiments, field captured *Hyalesthes obsoletus* were caged on differently treated *Catharanthus roseus* for five days*.* The insecticides lambda-cyhalothrin, deltamethrin, esfenvalerate, acetamiprid and chlorpyriphos showed the most rapid impact on insect survival and fully prevented phytoplasma transmission. The particle film forming products kaolin and diatomaceous earth had some effect. A transfer of the promising laboratory results to potato fields, however, was achieved to a limited extent only. Treatments with pyrethroids and acetamiprid every 8–10 days over the flight period of *H. obsoletus* roughly halved the number of symptomatic plants and tubers in case of moderately susceptible varieties and moderate infection pressure. In the event of susceptible varieties and high disease pressure, treatment effects were hardy discernible. In practical terms, the experiments indicate that insecticide applications alone are not sufficient to mitigate the disease. Spraying of diatomaceous earth and mineral oil did not affect disease incidence in the field.

**Supplementary Information:**

The online version contains supplementary material available at 10.1007/s41348-023-00768-y.

## Introduction

*‘Candidatus* Phytoplasma solani’ (Quaglino et al. 2013), also known as Stolbur phytoplasma, is a pathogen affecting a wide range of cultivated and wild plant species. Diseases such as Bois noir (BN) of grapevines, stolbur of potatoes and many vegetable species, maize redness and lavender decline are associated with this agent. The phytoplasma is prevalent all over Europe and the Mediterranean area (COST FA 0807 2014; CABI 2022).

The spread of the pathogen is linked to certain plant species, which serve both as phytoplasma reservoir and as developmental hosts for the transmitting insect species. Development of vector instars on infected host plants results in infectious vector adults, which transmit the pathogen to various crops. Studies in different regions of Europe have identified several distinct epidemiological cycles, each of them associated with specific plant hosts and insect vectors. So far, the Cixiid *Hyalesthes obsoletus* is considered as the principal vector species. In central Europe, from the middle of the twentieth century onwards, field bindweed (*Convolvulus arvensis*) acted as dual host for vector reproduction and for the pathogen (Brčák 1979; Maixner et al. 1995), whereas in Italy *Urtica dioica* was the relevant reservoir plant species (Lessio et al. 2007). During the last 15–20 years, however, *U. dioica* associated *H. obsoletus* populations were oftentimes also detected in central Europe (Jović et al. 2019). In France, *H. obsoletus* exploits lavender as developmental host and transmits specific ‘*Ca*. P. solani’ strains inducing lavender decline (Sémétey et al. 2018). In addition, *H. obsoletus* populations associated with *Vitex agnus-castus* and *Crepis foetida* were reported in South-East Europe (Kosovac et al. 2016, 2018, 2019). Several recent molecular studies indicate that distinct, genetically discernible *H. obsoletus* populations are specifically associated with the different host plant species (Johannesen et al. 2008; Kosovac et al. 2018; Jović et al. 2019 for review). In addition to *H. obsoletus*, several other Cixiidae planthoppers, as well as leafhoppers from the families Cicadellidae (Subfamilies Deltocephalinae, Agalliinae, Aphrodinae), Aphrophoridae and Delphacidae, are known or are likely to transmit the pathogen. These species might play some role in pathogen transmission or might be of local importance (Jović et al. 2019 for review; Quaglino et al. 2019; Riedle-Bauer et al. 2006, 2008; Battle et al. 2008).

Langer and Maixner (2004) observed that the ‘*Ca.* P. solani’ types specifically associated either with nettles or with bindweeds can be discriminated by analysis of the phytoplasma elongation factor *Tu* (*tuf)* gene. In the following years, a number of further ‘*Ca*. P. solani’ genes, such as *secY, stamp* and *vmp1,* have been characterised and found suitable for epidemiological studies. Nowadays, a fine tuned allocation of the phytoplasma types involved in a given disease outbreak to the respective disease cycle is possible with these marker genes (Cimerman et al. 2009; Fabre et al. 2011a, b; Johannesen et al. 2012; Aryan et al. 2014).

Past and recent scientific reports suggest a wide distribution of ‘*Ca* P. solani’ in Solanaceous crops in central and eastern Europe. E.g. stolbur outbreaks from 1921 to 1924 and from 1932 to 1934 in Slovakia seem likely and until the 1950 stolbur regularly occurred in former Czechoslovakia (Valenta 1953; Navrátil et al. 2009). At the same time, in Austria, Wenzl (1955) observed the outbreak of a “hairy sprout degeneration” of potatoes with incidences of up to 33% symptomatic potato plants in the field and up to 17.7% gummy tubers. More recently, namely around 2003, a severe stolbur outbreak affected celery production in North Eastern Italy (Trieste region, Carraro et al. 2008) and caused losses of up to 100%. In south Moravia, in 2006–2008 the pathogen severely damaged tomato production (Navrátil et al. 2009). A survey on widespread phytoplasma infections of potato crops in Romania and southern Russia in 2011 identified the stolbur phytoplasma as the major cause (Ember et al. 2011). From around 2016 onwards, the pathogen accounted for extreme losses in potatoes, vegetable crops and grapevines in various regions in East Austria. In potato, field symptoms included reddening and upward rolling of the top leaves and aerial tubers as well as wilting and withering of affected plants. Tubers were deformed, showed a soft and gummy consistency and developed hairy sprouts. Tomatoes produced discoloured, deformed leaves and greened flowers. Infected celery and Chinese cabbage plants turned yellow or red and declined (Brader and Riedle-Bauer, unpublished). Rates of symptomatic potato plants in the field reached up to 80%, and during stocking up to 40% of the harvested tubers proved unsaleable (Kamptner, personal communication). Phytoplasma infections caused up to 100% losses in celery fields as well as in tomato seed production. Molecular analyses of the *tuf* and *stamp* gene linked the involved phytoplasma types to bindweed related disease cycles. Extensive field observations revealed widespread bindweed associated populations of *H. obsoletus* in the affected regions (Brader and Riedle-Bauer unpublished). The presence of relevant insect populations on bindweed contrasted sharply to previous observations in Austria. In extensive vector monitorings related to BN outbreaks in grapevine from around 2003 onwards, bindweed associated *H. obsoletus* populations had been detected to a very limited extent only (Riedle-Bauer et al. 2006, 2008; Aryan et al. 2014; Johannesen and Riedle-Bauer 2014).

In general, management of phytoplasma diseases is challenging and in most cases conventional control strategies are not directly directed against the pathogen. Depending on the given situation, they focus on eradication of the infected reservoir plants and insecticide treatments against the vectors. In consequence, phytoplasma management could possibly have relevant economic and environmental implications (Bianco et al. 2019). In case of bindweeds, representing one of the principal sources for ‘*Ca* P. solani’, an eradication or extensive suppression is hardly feasible. This weed species can invade and persist in highly diverse habitats, so it is widespread not only in crop fields but also in their surroundings. Away from cultivated areas, in most cases, no weed control takes place. Within the fields and along field margins, vegetative reproduction through adventitious buds on roots, root parts and long-lived seeds complicate successful bindweed control. Any kind of mechanical disturbance, e.g. in the course of mechanical weeding or mowing works can aggravate the problem due to spreading of vegetative propagules (Davis et al. 2018).

The effect of conventional insecticides on phytoplasma spread is in general not well studied and a subject of controversial debate. Principally, in case of a vector transmittable disease, where infected vectors migrate into the crop sites, two aspects must be considered. One goal of the treatments is to keep vector populations inside the fields as low as possible and consequently reduce pathogen spread. Restriction of vector populations inside a crop, however, does not protect the first crop plant(s) reached by a vector insect migrating into the field. In this respect, insecticides actually preventing pathogen inoculation by disrupting the insect feeding behaviour in a shorter time than the minimum inoculation access period (IAP) are required. In earlier examinations, insecticide treatments reduced the spread of the pathogen within the crop but even frequent applications did not prevent the appearance of the disease (Weintraub 2007). Studies on psyllids as vectors of European Stone fruit yellows phytoplasma (‘*Candidatus* Phytoplasma prunorum’) suggested that some insecticide agents, particularly pyrethroids, act quickly and could therefore have a direct effect on phytoplasma transmission (Paleskić et al. 2017). This presumption is strengthened by the fact that insecticide treatments have been used for years to control the spread of apple proliferation (‘*Candidatus* Phytoplasma mali’) in Northern Italy (Baldessari et al. 2010; Österreicher and Unterthurner 2015). Šafářová et al. (2016) reported lower presence of psyllid species known as vectors of ‘*Ca*. P. mali’ and lower numbers of infected apple trees in orchards under integrated management as compared to organic management.

The recent Stolbur outbreak in Austria raised the question, to which extent pathogen spread could be restricted by insecticide or insect repellent treatments. This question was of interest on one hand for producers of consumer potatoes and vegetables but, particularly, for seed potato growers. Therefore, we studied the effects of various insecticides and insect deterrents and analysed their suitability for a future sustainable Stolbur management. Laboratory experiments focused on the effects of the test agents on mortality of *H. obsoletus* and on phytoplasma transmission. The most promising active compounds were included in a series of field experiments implemented over three years in potato. Treatment effects on vector density, disease symptoms on plants and tubers as well as on phytoplasma infections of tubers were investigated.

## Material and methods

### Laboratory experiments

#### Evaluation of plant protection products

The effects of a range of insecticides and insect deterrents on insect mortality and pathogen transmission were evaluated in pot cage experiments including *Catharanthus roseus* as test plants and field collected *H. obsoletus*. The experiments took place in June 2018–2020. *C. roseus* (cv. ‘Sorbas Reinweiß’, Austrosaat, Vienna, Austria), were grown from cuttings under laboratory conditions (22–24 °C, L:D 16:8) until six to eight leaf stage. Table 1 illustrates the tested agents, the manufacturers and the used concentrations. The concentrations of the test compounds were derived from the product registrations, namely the application rates, and the spray volume per hectare. The application of test compounds and the efficacy tests took place under outdoor conditions but protected from rain. Test plants received spraying until run off by aid of a 1.25 l hand sprayer (Birchmeier, Stetten, CH). In order to ensure even wetting, spraying started at the uppermost leaves working towards the base. The spraying residues were allowed to air dry for two to four hours. In case of acetamiprid, additional tests with four-day-old spraying residues in case of lambda cyhalotrin and flupyradifuron tests with five-day-old spraying residues were also included in the study (“aged residues”, Table 1). Each test plant was covered by a transparent cylindrical cage (diameter 10 cm, length 15 cm). *H. obsoletus* were collected in the field at four locations in Weinviertel (Lower Austria) on bindweeds by vacuum sampling (Online resource 1). They were transported to the laboratory within at maximum four hours under cooled conditions. Per cage, five *H. obsoletus* individuals were released. Insect mortality in each cage was recorded after one hour, three hours, 24 h, two, three and five days. After five days, the remaining individuals were removed from the test plants and rates of phytoplasma–positive insects were determined by PCR analysis. The test plants were transferred back to the laboratory, cultivated for five to six weeks, inspected for disease symptoms and analysed by PCR. All tests were repeated seven to eight times (seven to eight independent experiments each including one test plant and five insects caged on it). Water-treated plants served as controls.

**Table 1:** Compounds included in the laboratory study

Insecticide	Active ingredient	Abbreviation	Conc.% (w/v) or (v/v)	Manufacturer
Sumi Alpha	Esfenvalerate	Esfenval	0.1	FMC Agro, Graz, A
Benevia	Cyantraniliprole	Cyantran 0.04	0.04	FMC Agro
Benevia	Cyantraniliprole	Cyantran 0.25	0.25	FMC Agro
Closer	Sulfoxaflor	Sulfoxa	0.13	Kwizda, Vienna, A
Control	water	Control water		
Cutisan	Kaolin clay	Kaolin	4	Biohelp, Vienna, A
Decis	Deltamethrin	Deltamethr	0.025	Bayer, Vienna, A
Karate Zeon	Lambda-Cyhalothrin	L Cyhal	0.025	Syngenta, Vienna, A
Karate Zeon 5d aged residue	Lambda-Cyhalothrin	L Cyhal 5d	0.025	Syngenta
Mospilan 20 SG	Acetamiprid	Acetami 0.033	0.033	Kwizda
Mospilan 20 SG	Acetamiprid	Acetami 0.08	0.08	Kwizda
Mospilan 20 SG 4d aged residue	Acetamiprid	Acetami 0.033 4d	0.033	Kwizda
Mospilan 20 SG 4d aged residue	Acetamiprid	Acetami 0.08 4d	0.08	Kwizda
Movento	Spirotetramate	Spirotet	0.0025	Bayer
Neem	Azadirachtin		0.8	Biohelp
Quassia	*Quassia amara*	Quassia	0.0045	Biohelp
Reldan	Chlorpyriphos	Chlorpyri	0.3	Kwizda
Silicosec	Silicate powder (diatomaceous earth)	Diatom.earth	4	Biohelp
Sivanto	Flupyradifuron	Flupyradi	0.125	Bayer
Sivanto aged residue	Flupyradifuron	Flupyradi 5d	0.125	Bayer
Spin Tor	Spinosad	Spino		Biohelp
Teppeki	Flonicamid	Flonica	0.0530	Belchim, Schwechat, A

#### PCR analysis

DNA extraction from *C. roseus* and *H. obsoletus* was carried out by a CTAB procedure as described previously (Maixner et al. 1995). Each plant or insect was analysed individually. In case of leaf samples, each sample consisted of midrib and petiole tissue of three leaves.

DNA extraction from potato tubers was carried out by a Nucleon Phytopure genomic DNA extraction kits according to the protocol of the manufacturer (GE Healthcare, UK). For each tuber, four 10 × 5 × 5 mm sized pieces including skin of washed potatoes were cut and ground in liquid nitrogen with pestle and mortar. 0.1 g of the obtained powder was used for extraction in 1.5 mL tubes with half amounts as in the protocol and with an additional RNAse step (1 µl of 5 mg/mL DNAse and Proteinase free RNAse from Thermo Fisher Scientific (UK) for 30 min at 37 °C) after cell lysis. Plant samples were analysed for ‘*Ca.* P. solani’ by a nested PCR procedure including the primers P1/P7 (Deng and Hiruki 1991; Schneider et al. 1995) and STOLF/STOLR (Maixner et al. 1995) or using the nested *Stamp* protocol using (Stamp-F, Stamp-R0, Stamp-F1 and Stamp-R1; Fabre et al. 2011b). Phytoplasma presence in *H. obsoletus* was determined by real time PCR following the method described by Christensen et al. (2004).

### Treatment effects in the field

In principle, field experiments were carried out in Rottersdorf, Maissau, Unterhautzenthal and Oberfellabrunn in Lower Austria in commercial potato farms in three consecutive years (2019–2021). However, in Unterhautzenthal 2019 and 2020, there were no signs of the disease and no *H. obsoletus*, in Oberfellabrunn in 2019, severe drought stress impeded the evaluation of the experiment. In consequence, the current paper focuses on our experiments in Maissau 2019–2021 and in Rottersdorf 2019 and 2020. The linear distance of the two test sites is about 34 km. The distance at on site but between the different years are less than 1 km. Details on the experimental fields and the spraying conditions are outlined in Table 2. Due to the suspected rapid movement of *H. obsoletus* in the fields, we considered large treatment plots as necessary. Moreover, the farms were equipped with standard spraying devices only. Therefore, we opted for experiments in a strip design including two repetitions per treatment, location and year, except for Maissau 2021 where due to the shape of the field 2 × 2 blocks were laid out (Online resources 2–6). The length of the fields varied from 275 to 330 m, the plot width was adjusted to the spray width of the respective field sprayer (27 m in Maissau, 28 m in Rottersdorf,). The included insecticides were selected according to the results of the laboratory experiments and a given registration of the respective compound for use in potato. In addition, treatments with diatomaceous earth and a paraffin oil were included in the experiments. The start of the treatments was determined according to the *H. obsoletus* monitoring carried out by aid of vacuum sampling at roadsides and field margins at several locations in Southern Weinviertel (Naglern1, Naglern 2, Weinsteig, Lower Austria, outlined in Online resource 1). The first application was scheduled one week after the first specimen were detected.

**Table 2:** Description of the potato farms and their equipment

	Potato farm 1	Potato farm 2
Location	Maissau/Lower Austria/Austria	Rottersdorf/Lower Austria/Austria
GPS	Field 2019: 48.569543, 15.836777; Field 2020: 48.572211, 15.838700; Field 2021 48.567704, 15.839625;	Field 2019: 48.306569, 15.606075; Field 2020: 48.250649, 15.634724;
Sprayer	Kverneland XTrack C40 (Klepp, N) 4000 l capacity	Amazone UX 6200 Super (Amazone, Hasbergen, D)
Nozzle	Lechler IDKT 03 (Lechler, Metzingen, D)	Insecticides: Agrotop Turbo Drop HiSpeed 110–04 Standard TD, (Agrotop, Obertraubling, D) working pressure 3 bar Diatomaceous earth: Lechler compact double flat fan nozzle IDKT 120–05; working pressure 5 bar
Driving Speed	8 km/h	Insecticides: 9,8 km/h, diatomaceous earth: 7.5 km/h
Variety	2919, 2021 Eurostarch, 2020 Nafida	2019 Tosca; 2020 Belmonda
Volume spray liquid [l/ha]	200	Ins 2019 + 2020: 200 l; Diatom. Earth 2019: 400 l

#### Experiments 2019

In Maissau (Online resource 2) treatments started on June 13, the included potato variety was ‘Eurostarch’. The farmer decided to apply six treatments against *H. obsoletus*, the last application was on July 16 (Table 3). For determination of field symptoms in each plot the first 50 plants in rows six and seven on the left hand side of the driving lane were visually classified into the categories healthy (0) and symptomatic (1). Visual inspections were carried out on July, 17, July 25 and August 19. At harvest, in each case circa100 tubers per plot were collected both from the field edge and from the centre of the field. On October 15, 100 tubers per experimental plot both from the field edge and the centre of the field were analyzed visually and by squeezing with fingers and classified into the categories healthy (0) and symptomatic (1). Every tenth tuber was collected for PCR analysis.

**Table 3:** Treatments, applied compounds and dates of application at the test site Maissau 2019–2021

Year	Treatment	Compound	Application rate	Date of application
2019	Insecticide (Ins)	Esfenvalerate (FMC Agro, Graz, A) Silwet Top (wetting agent: BASF, Vienna, A)	0.2 l/ha 0.1 l/ha	13.6., 24.6., 5.7., 16.7
		Acetamiprid (Kwizda, Vienna, A; against *Leptinotarsa decemlineata*) Silwet Top	0.25 kg/ha 0.1 l/ha	15.6
		Lambda-Cyhalothrin (Syngenta, Vienna, A) Silwet Top	0.075 l/ha	18.6., 28.6
	Mineral oil (Min)	White mineral oil (EMU 11E, Star Agro, Allerheiligen bei Wildon, A) Silwet Top	1 l/ha 0.1 l/ha	Every 5 days from 13.6.-16.7
		Acetamiprid (against *L. decemlineata*)	0.25 kg/ha	15.6
	Control	Acetamiprid (against *L. decemlineata*)	0.25 kg/ha	15.6
2020	Ins	Lambda-Cyhalothrin Acetamiprid Silwet Top	0.075 l/ha 0.25 kg/ha 0.1 l /ha	23.6
		Cypermethrin (Cymbigon Forte, Kwizda) Silwet Top	0.05 l /ha 0.1 l/ha	30.6
		Lambda-Cyhalothrin Acetamiprid Silwet Top	0.075 l/ha 0.1 kg/ha 0.1 l /ha	4.7
		Cypermethrin Silwet Top	0.05 l /ha 0.1 l/ha	13.7
		Esfenvalerate Silwet Top	0.2 l /ha 0.1 l/ha	21.7
	Control			
2021	Ins	Chlorantraniliprole (Coragen, FMC Agro, Graz; A; against *L. decemlineata*) Silwet Top	0.06 l/ha 0.01 l/ha	21.6
		Esfenvalerate Acetamiprid Silwet Top	0.2 l/ha 0.1 kg/ha 0.1 l /ha	2.7
		Lambda-Cyhalothrin Silwet Top	0.075 l/ha 0.1 l /ha	12.7
		Esfenvalerate Acetamiprid Silwet Top	0.2 l/ha 0.25 kg/ha 0.1 l /ha	21.7
		Lamba-Cyhalothrin Silwet Top	0.075 l/ha 0.1 l /ha	29.7
	Control	Chorantraniliprole (against *L.decemlineata*) Silwet Top	0.06 l/ha 0.01 l/ha	21.6

In Rottersdorf, the test field (Online resource 3) was planted with the variety ‘Tosca’. The first application out of four was on June 13 and the last one on July 5 (Table 4). Visual inspections on July 16, July 31 and August 21 were carried out as described for Maissau, except that only 40–42 plants per row (rows six and seven on the left-hand side of the driving lane) were inspected. On October 1, the harvest of each treatment (both repetitions together) was transferred into four distinct bulk bins. Tuber analysis (100 tubers per bin) and sampling for PCR analysis were carried out on October 18 as described above.

**Table 4:** Treatments, applied compounds and dates of application at the test site Rottersdorf 2019–2020

Year	Treatment	Compound	Concentration	Date of application
2019	Ins	Esfenvalerate Silwet Top	0.2 l/ha 0.15 l/ha	13.6., 28.6
		Lambda-Cyhalothrin Silwet Top	0.075 l/ha 0.15 l/ha	18.6., 5.7
	Diatom.earth	Diatomaceous earth (Silicosec Biohelp, Vienna A) Prev AM (wetting agent, Biohelp)	20 kg/ha 0.4% (v/v)	13.6., 18.6., 28.6., 5.7
	Control	No insecticide treatment		
2020	Ins	Lambda-Cyhalothrin Acetamiprid Designer (wetting agent, Kwizda)	0.075 l/ha 0.25 l /ha 0.15 l /ha	19.6
		Cypermethrin Designer	0.05 l /ha 0.15 l/ha	27.6., 10.7
		Lambda-Cyhalothrin Acetamiprid Designer	0.075 l/ha 0.01 l /ha 0.15 l /ha	2.7
		Esfenvalerate Designer	0.2 l /ha 0.15 l/ha	15.7
	Control	No insecticide treatment		

At both locations, from the beginning of the treatments onwards, the presence of *H. obsoletus* in the field was monitored by yellow sticky traps (25 × 40 cm, Horiver, Biohelp, Vienna, A) mounted in the rows transversely to the direction of the rows. The traps were installed at a distance of 5 m and 20 m from the field edge. Per plot and distance from the field edge, two traps were installed and changed in five to seven day intervals (in total 24 traps per time interval; Online resources 2 and 3).

#### Experiments 2020

In Maissau the experimental field (Online resource 4) was planted with the variety ‘Nafida’. Treatments started on June 23, until July 21, five treatments were applied (Table 3). Visual inspections on July 31 and August 14 revealed considerably lower infection rates than in 2019, therefore the number of inspected plants in each plot was increased to 240. At harvest on October 1, 200 tubers per plot were randomly collected for analysis as described for 2019. A separate harvest of tubers from the field margin and the middle of the field was not maintained as no difference had been recorded in 2019.

In Rottersdorf (Online resource 5), the experimental field was planted with the variety ‘Belmonda’. The treatments started on June 19, until July 15 in total five applications were carried out (Table 4). Visual inspections in the field took place as described on July 30 and on August 14. Tubers were harvested on October 6 and investigated on Oktober 21 as described for 2019. Every tenth tuber was collected for PCR-analysis.

Numbers of yellow sticky traps per sampling interval were reduced to five in Maissau and four in Rottersdorf and mounted as outlined in Online resources 4 and 5.

#### Experiments 2021

The experiments took place in Maissau only and included the variety ‘Eurostarch’. The first treatment was applied on June 21. In total four treatments directed against stolbur vectors were carried out, the last one on July 29 (Table 3). Per plot, one yellow sticky trap was mounted in a distance of 20 m from the field edge and changed weekly (Online resource 6). Visual analysis of the plants in the field was carried out as described on July 31 and August 19. The experimental plots were harvested on October 11. Due to the low incidence of field symptoms, the number of analysed tubers was increased to 400 per experimental plot. The analysis of the tubers took place as described for the previous years on December 29. At the same time, every tenth tuber was collected for subsequent PCR analysis.

### Statistical analyses

All statistical analyses were performed individually for each test year and location by aid of the statistics program SPSS 26.0 (SPSS, IBM, Vienna, A). The effect of the treatments on insect survival in the laboratory was studied by aid of generalized linear models including insect survival (dead = 1, alive = 0) as the outcome, the model type binary logit, and the explanatory variable tested compound. We considered the factor hours post exposure as crucial for the subsequent choice of compounds for the field experiments. Therefore, individual models were run for all selected exposure durations instead of including duration of exposure as a factor in a joint model (Table 5).

**Table 5:** Statistical analysis of laboratory experiments on insect mortality

	Duration of exposure			
	1 h	3 h	24 h	3d
Statistical model, factor: treatment	Wald *χ*^2^ = 202.51 df = 21; *p* = 0.000	Wald *χ*^2^ = 90.53, df = 20; *p* = 0.000	Wald *χ*^2^ = 62.17, df = 21; *p* = 0.000	Wald *χ*^2^ = 149.55, df = 20; *p* = 0.000
Estimated marginal mean				
Acetami 0.033%	**0.63**	**1.00**	**1.00**	**1.00**
Acetami 0.033% 4d	**0.77**	**1.00**	**1.00**	**1.00**
Acetami 0.08%	**0.80**	**1.00**	**1.00**	**1.00**
Acetami 0.08% 4d	**0.75**	**1.00**	**1.00**	**1.00**
Azadirach	**0.25**	**0.42**	**0.72**	**0.82**
Chlorpyri	**0.27**	**0.50**	**1.00**	**1.00**
Cyantran 0.04	0.05	0.10	0.45	n.a
Cyantran 0.25	0.10	0.25	0.40	**0.75**
Deltamethr	**0.92**	**0.90**	**1.00**	**1.00**
Diatom.earth	0.20	0.22	0.30	0.27
Esfenval	0.30	n.a	**0.57**	1.00
Flonica	**0.30**	**0.30**	**0.42**	**0.80**
Flupyradi	**0.80**	**0.97**	**1.00**	**1.00**
Flupyradi 5d	**0.47**	**0.65**	**0.85**	**0.95**
Kaolin	**0.30**	0.27	0.35	0.40
L-Cyhal	**1.00**	**1.00**	**1.00**	**1.00**
L-Cyhal 5d	**0.70**	**0.80**	**1.00**	**1.00**
Quassia	**0.30**	**0.32**	0.32	**0.72**
Spino	**0.27**	**0.31**	**0.46**	**0.80**
Spirotet	0.09	0.17	0.17	0.29
Sulfoxa	**0.54**	**0.92**	**1.00**	**1.00**
Control water	0.10	0.12	0.24	0.29

Values printed in bold differ significantly from water treated control (*p* ≤ 0.05); *n.a.* not assessed

To identify treatment effects on the presence of disease symptoms on potato plants in the field and on potato tubers, we calculated generalized linear models for the response variable presence of disease symptoms (symptomatic = 1, healthy = 0). The model type binary logit, and the categorical explanatory variables (1) treatment, (2) date of scoring (in case of plants in the field only, not for tubers), (3) position within the layout of the experiment (position 1, position 2, except for analysis of tubers in Rottersdorf, online resources 2–6) and (4) area of the field from which the tubers had been harvested (from middle or edge of the field, for Maissau 2019 only) were included (Tables 6 and 7).

**Table 6:** Statistical analysis of the experiments in Maissau 2019–2021. Different letters indicate statistically significant differences in pairwise comparisons (*p* ≤ 0.05)

Description of model				
Dependant variable/ Factors	Wald χ^2^	p	df	Pairwise comparison (LSD) of treatments (estimated marginal means)
*Maissau 2019*				
Visual scoring field				
Treatment	51.98	0.000	2	Ins (0.19)^a^, Min (0.32)^b^ Control (0.38)^c^
Scoring date	211.85	0.000	2	July 17 (0.13)^a^ Jul 25 (0.27)^b^, Aug 19 (0.53)^c^
Position	0.15	n.s	1	
Visual scoring tubers				
Treatment	7.81	0.02	2	Ins (0.19)^a^, Control (0.25)^b^, Min (0,27)^c^
Position within the experimental design	0.435	n.s	1	
Area of the field	1.067	n.s		
Insect capture on sticky traps				
Treatment	0.45	n.s	2	
Distance to field edge	0.41	n.s	1	
Date of trap removal	55.00	0.000	4	
*Maissau 2020*				
Visual scoring field				
Treatment	9.47	0.002	1	Ins (0.01), Control (0.01)
Scoring date	18.61	0.000	1	July 31 (0.00), August 14 (0.04)
Position within the experimental design	0.02	n.s	1	
Visual scoring tubers				
Treatment	0.036	n.s		
*Maissau 2021*				
Visual scoring tubers				
Treatment	5.02	0.025		Ins (0.03) Control (0.05)
Position within the experimental design	7.39	0.007		Pos1 1 (0.3), Pos 2 (0.5)

**Table 7:** Statistical analysis of the experiments in Rottersdorf 2019–2020. Different letters indicate statistically significant differences in pairwise comparisons (p ≤ 0,05)

Description of model				
Dependant variable/factors/	Wald χ^2^	*p*	df	Pairwise comparison (LSD) of treatments (estimated marginal means)
Rottersdorf 2019				
*Visual scoring field*				
Treatment	48.56	0.000	2	Ins (0.30)^a^ Diatom.earth (0.46)^b^ Control (0.55)^c^
Scoring date	369.53	0.000	2	Jul 16 (0.15)^a^, Jul 31 (0.37)^b^, Aug 21 (0.82)^c^
Position within the experimental design	7.75	0.005	1	Pos 1 (0.39), Pos 2 (0.48)
Visual scoring tubers				
Treatment	3.61	n.s	2	
Insect capture on sticky traps				
Treatment	10.54	0.005	2	Ins (0.66)^a^, Control (0.98)^b^, Diatom. Earth (1,12)^c^
Distance to field edge	20.13	0.000	1	20 m (0.67), 5 m (1.21)
Date of trap removal	59.91	0.000	3	
*Rottersdorf 2020*				
Visual scoring field				
Treatment	5.73	0.018	1	Ins (0.11)^a^ Control (0.16)^b^
Scoring date	54.69	0.000	1	Jul 30 (0.04), Aug 14 (0.20)
Position within the experimental design	26.57	0.000	1	Pos 1(0.06), Pos 2 (0.15)
Visual scoring tubers				
Treatment	6.49	0.011	1	Ins (0.033)^a^ Control (0.075)^b^

In 2019, insect counts on yellow sticky traps were analysed by aid of generalized Poisson models comprising the response variable number of *H. obsoletus* per trap and the categorical explanatory variables (1) treatment, (2) sampling period and (3) distance from the edge of the field (5 m versus 20 m).

We tested for the main effects as offered by the programme. Where appropriate, least significant difference (LSD) tests were calculated to analyse differences among the effects of the studied explanatory variables. For the LSD tests a 0.05 level of significance was applied.

## Results

### Evaluation of plant protection agents under laboratory conditions

**Fig. 1:**
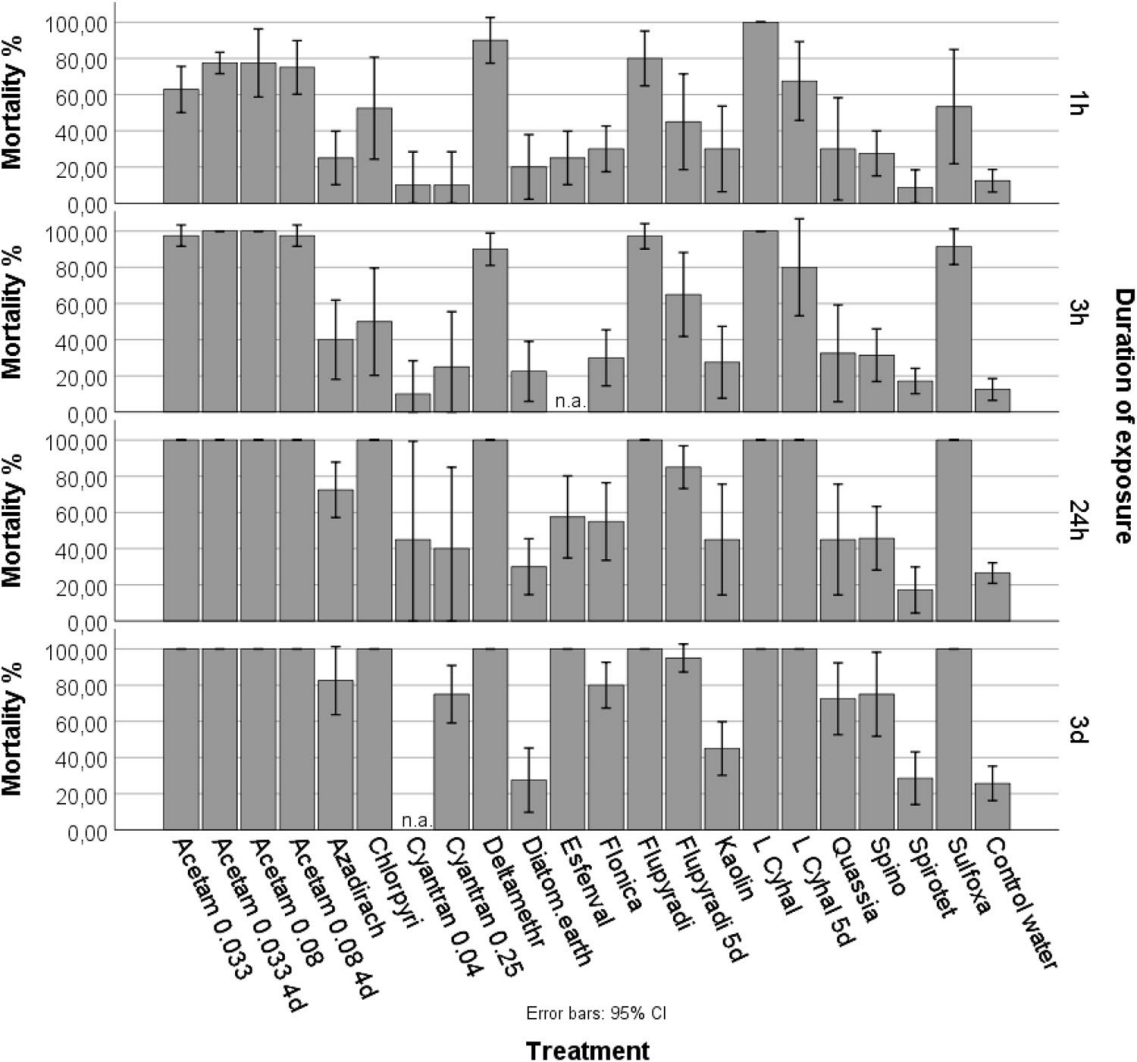
Insect mortality in laboratory experiments 1 h, 3 h, 24 h and 3 days after start of the experiments; n.a. not assessed

Already one hour after insect release a clear and statistically significant treatment effect on insect survival was recorded (Wald *χ*^2^ = 360.65, *df* = 21; *p* = 0.00). All tested compounds significantly reduced insect survival as compared to the control, except cyantraniliprole 0.04%, cyantraniliprole 0.25%, spirotetramate and diatomaceous earth. Lambda-cyhalothrin and deltamethrin were significantly more effective than all other tested compounds. Flupyradifuron, acetamiprid 0.08% and acetamiprid 0.033% also proved highly effective. After three hours of exposure, in addition to lambda-cyhalothrin and deltamethrin an insect mortality at or near to 100% was observed for the compounds acetamiprid (for both concentrations), sulfoxaflor and flupyradifuron. Five-day-old residues of lambda-cyhalothrin and four-day-old residues of acetamiprid in both concentrations still significantly enhanced mortality as compared to the untreated control (Fig. 1, Table 5).

**Fig. 2:**
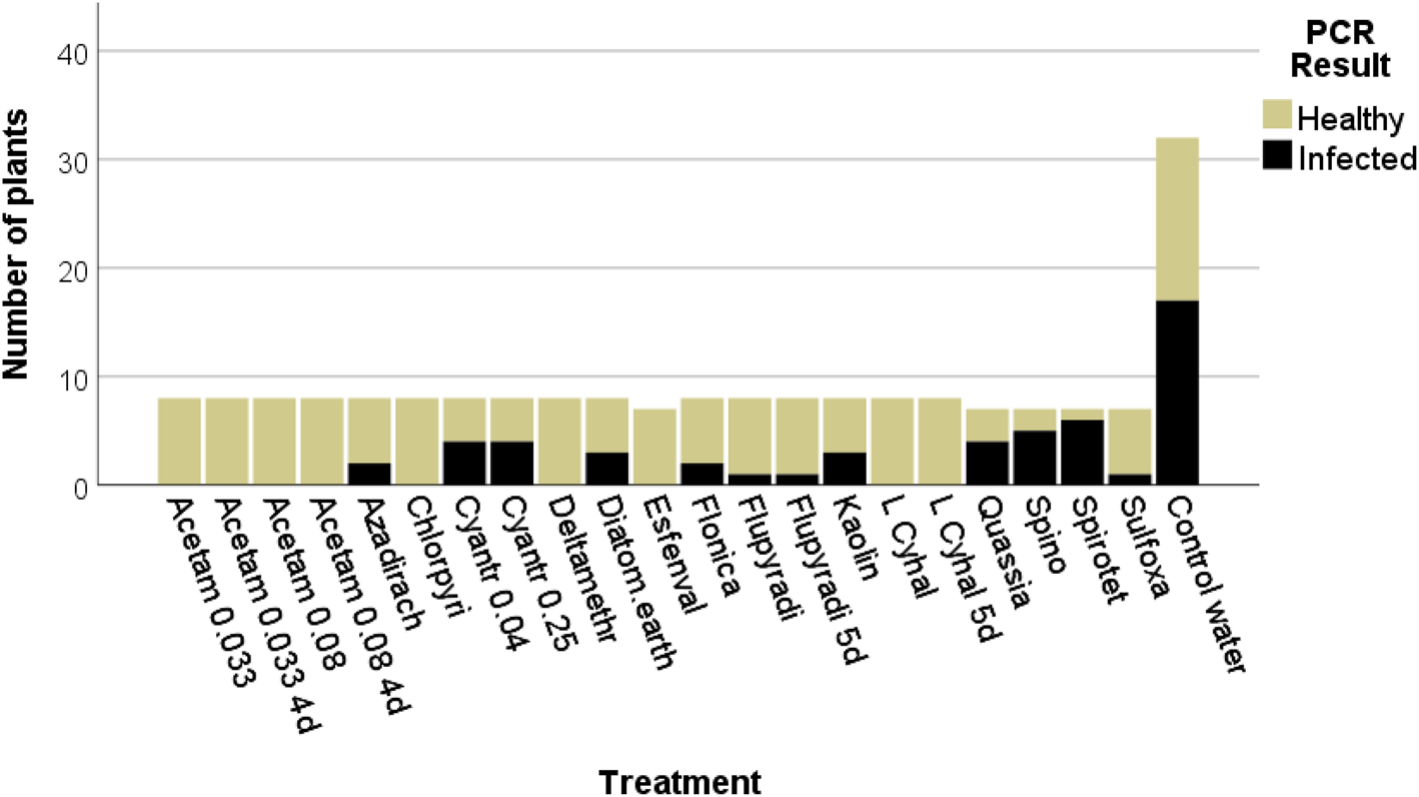
PCR analysis of the *C. roseus* test plants from the laboratory insecticide experiments

All *C. roseus* plants included in the laboratory experiments were analysed for phytoplasma infection by PCR with primers P1/P7 and StolR/StolF after the end of the experiment. As illustrated in Fig. 2, all test plants treated with lambda-cyhalothrin, deltamethrin, acetamiprid, chlorpyriphos and esfenvalerate remained healthy, whereas 53% of the control plants became infected. The highest infection rates were recorded for quassia (57%), spinosad (71%) and spirotetramate (86%). Out of the test plants treated with particle films (kaolin, diatomaceous earth) 37% were found infected. All PCR positive plants developed classical phytoplasma symptoms such as flower greening, lack of flowers, yellow, downsized leaves and growth reduction. Q-PCR analysis of the insects included in the transmission tests revealed phytoplasma infection rates of 33–50% depending on the sampling site (Online resource 1).

### Field experiments

#### Maissau 2019

**Fig. 3:**
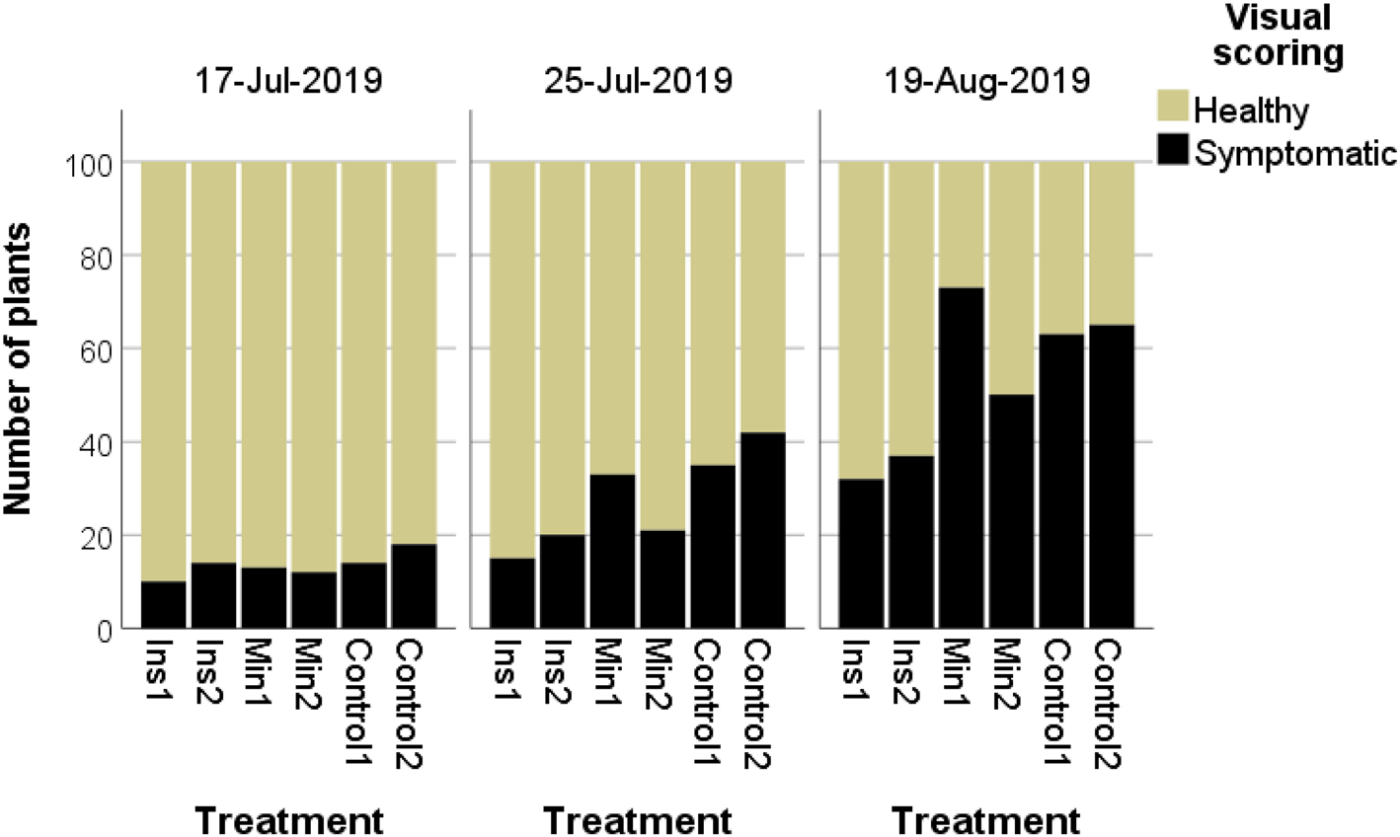
Maissau 2019: Rates of visually diseased plants in the field in the course of the experiment. Applied compounds: in all treatments: Acetamiprid against *L. decemlineata*; Ins: Insecticides: Esfenvalerate, Lambda-Cyhalothrin, Min: White mineral oil

On July 17, 10.0% and 14.0% of the plants in the insecticide treated plots, 12.0% and 13.0% of the plants in the mineral oil treated plots and 14.0% and 18.0% of the plants in the control plots were symptomatic. On July 25, the infection rates rose constantly to reach 15.0% to 20.0% of symptomatic plants in the insecticide treated plots, 21.0% to 34.0% in the mineral oil treated plots and 36.0% to 42.0% in the control plots. On August 19, the control plots showed infection rates between 63 and 65%, in the insecticide plots 32% and 37%, in the mineral oil plots 73% and 50% of the plants were visually diseased (Fig. 3). Generalized linear models indicated a significant effect of the factors treatment and scoring date (treatment: Wald *χ*^2^ = 51.98.08. *df* = 2, *p* = 0.000; scoring date: Wald *χ*^2^ = 211.85, *df* = 1, *p* = 0.000) but no effect of the factor position within the experimental plot. Pairwise comparisons of the treatments revealed a significantly lower rate of symptomatic plants in the insecticide treatments, whereas mineral oil had no effect as compared to the untreated control. Pairwise comparisons of scoring dates showed a significant increase of diseased plants in the course of the experiment (Table 6).

Rates of symptomatic tubers in the insecticide treatments (20.0% and 18.6% at the field edge, 20.6% and 14.9% in the middle of the field, respectively) were somewhat lower than in the control plots (28.4% and 26.0% at the field edge, 22.5% and 23.5% in the middle of the field) and the mineral oil plots (31.1% and 23.1% at the field edge, 27.5% in the middle of the field). Statistical analysis indicated a significant influence of the factor treatment (Wald *χ*^2^ = 7.81, *df* = 2, *p* = 0.02) but no effect of the factors position within the experimental field and area in the field (edge, middle), from which the tubers had been harvested (Table 6, Online resource 7). PCR analysis of the tubers by nested Stamp PCR revealed an average infection rate of 30% for the control treatment, 22.5% for the insecticide treatment and 50% for the diatomaceous earth treatment.

**Fig. 4:**
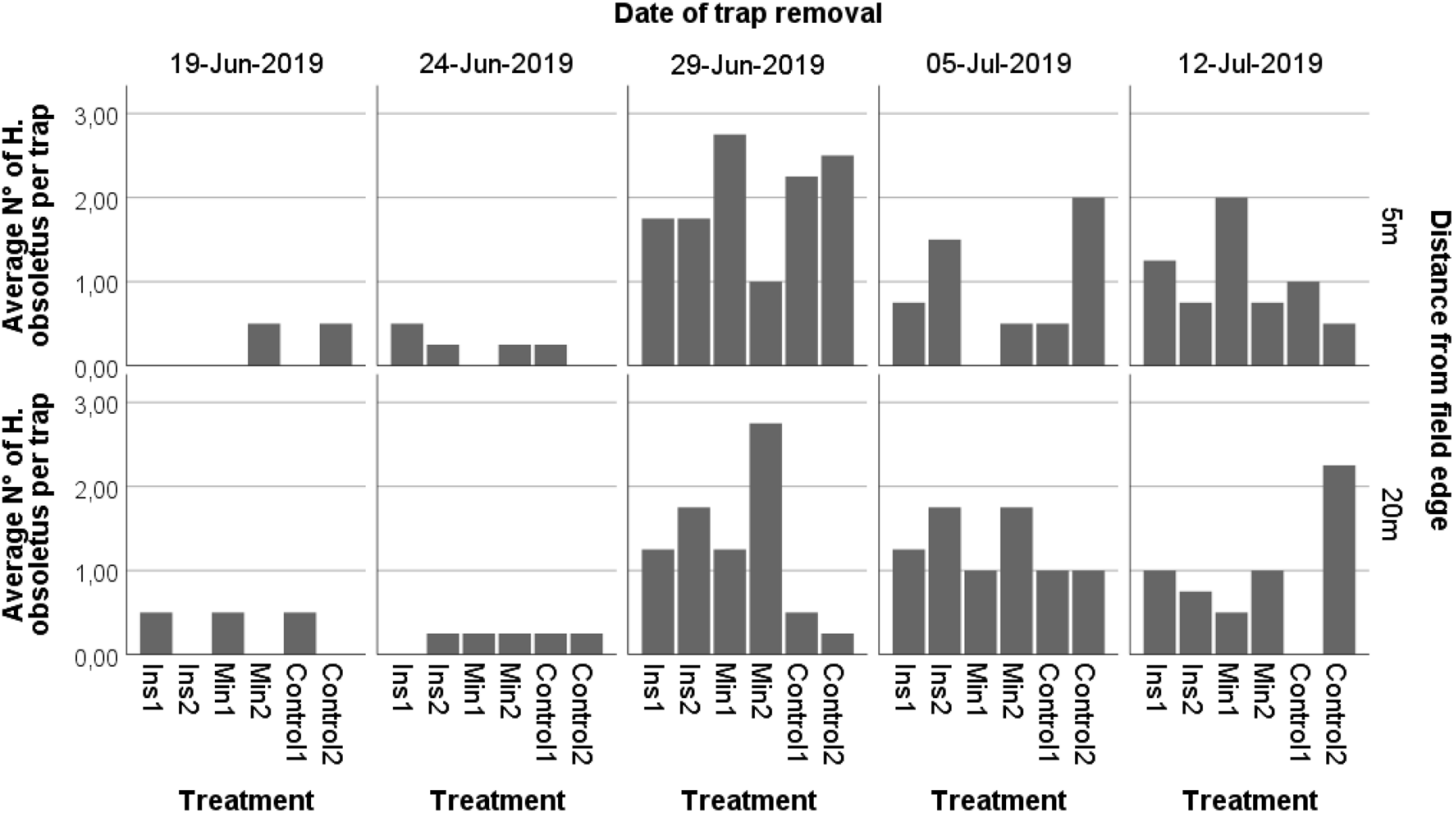
Maissau 2019: Insect captures on yellow sticky traps. Applied compounds: in all treatments: Acetamiprid against *L. decemlineata*; Ins: Insecticides Esfenvalerate, Lambda-Cyhalothrin, Min: White mineral oil

Analysis of planthoppers in the field by yellow sticky traps showed a relevant insect density from the sampling period June24–June 29 onwards. Statistical analysis indicated a significant effect of the sampling date (Wald *χ*^2^ = 55.00, *df* = 4, *p* = 0.000) on insect numbers on the traps but no effect of the treatment and the distance to the field edge at which the yellow sticky traps had been mounted (Table 6, Fig. 4).

#### Rottersdorf 2019

**Fig. 5:**
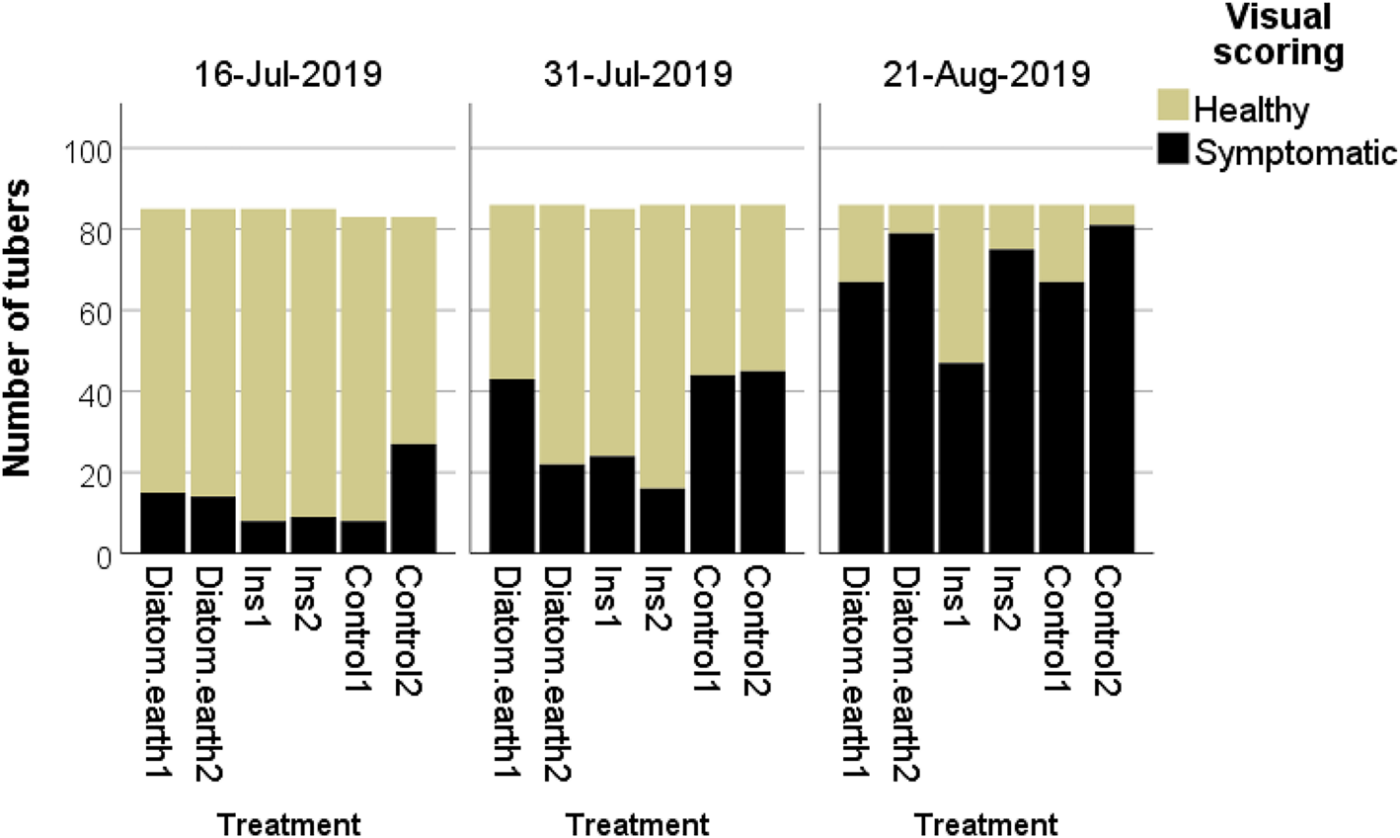
Rottersdorf 2019: Rates of visually diseased plants in the field in the course of the experiment. Applied compounds: Ins: Insecticides: Esfenvalerate, Lambda-Cyhalothrin; Diatom.earth: Diatomaceous earth

At the visual inspection on July 16, rates of symptomatic plants in the insecticide plots were 9.4% and 10.6%, respectively, in the diatomaceous earth plots 17.6% and 16.5%, in the control plots 9.6% and 32.5%. On July 31, infection rates in the insecticide plots had risen to 28.2% and 18.6%, in the diatomaceous earth plots to 50.0% and 25.6%, and in the control plots to 51.2% and 52.3%. At the last visual inspection (August 21), infection rates of all plots exceeded 75%, except the plot Ins1 with a rate of 54.7% of symptomatic plants. The generalized linear model indicated a significant effect of the factors treatment, scoring date and position within the experimental design on rates of symptomatic plants (Treatment: Wald *χ*^2^ = 48.56. *df* = 2, *p* = 0.000; scoring date: Wald *χ*^2^ = 369.53, df = 2, *p* = 0.000; position: Wald *χ*^2^ = 7.75 *df* = 1, *p* = 0.000; Fig. 5, Table 7).

The treatments had no effect on the health status of the tubers (Online resource 8, Table 7). Rates of symptomatic tubers of 17.0 and 26.0% were recorded for the control plots, 14.0 and 24.0% for the diatomaceous earth plots, 13.0 and 21.0% for the insecticide plots. PCR analysis confirmed the low treatment effect on the health status of the tubers. Rates of positive tubers with nested Stamp PCR were 57.7%, 82.5% and 65.0% for the insecticide treatment, the diatomaceous earth treatment and the experimental control, respectively.

**Fig. 6:**
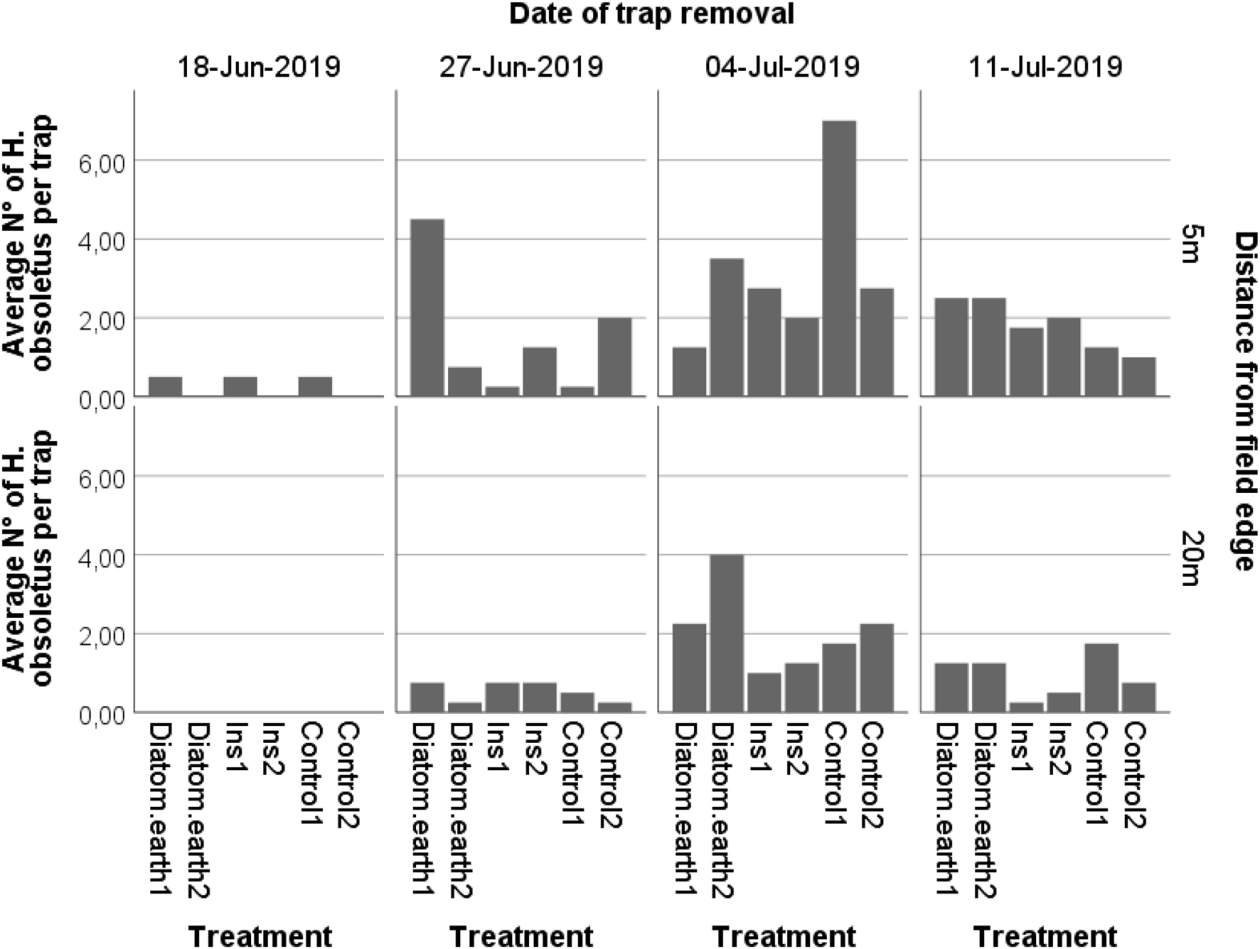
Rottersdorf 2019: Insect captures on yellow sticky traps. Applied compounds: Ins: Insecticides: Esfenvalerate, Lambda-Cyhalothrin; Diatom.earth: Diatomaceous earth

The highest number of *H. obsoletus* were captured during the sampling interval June 27-July 4. The factors treatment, sampling date and the distance to the field edge at which the yellow sticky traps had been installed significantly affected insect counts (Treatment: Wald χ2 = 10.54, df = 2, p = 0.000; sampling date: Wald χ2 = 59.91, df = 3, p = 0.000; distance to field edge Wald χ2 = 20.13 df = 1, p = 0.000; Fig. 6, Table 7).

#### Maissau 2020

Symptoms ascribable to ‘*Ca*. P. solani’ increased from single plants on July 31 to a disease incidence of 0.8% and 4.2% in the insecticide plots and 8.3% and 4.6% in the control plots two weeks later. The generalized linear model proved significant impacts of the factors treatment and scoring date on rates of symptomatic plants in the field. (Treatment: Wald *χ*^2^ = 9.74, *df* = 1, *p* = 0.002; scoring date: Wald *χ*^2^ = 18.61, *df* = 1, *p* = 0.000), but no effect of the position of the plot on the field (Online resource 9, Table 6).

Visual assessment of tubers after 4 month of storage showed no significant difference between treatments, rates of 3.0% and 4.0% of soft tubers in insecticide versus 3% and 4% in control treatments were recorded (Online resource 10). PCR using nested *Stamp* primers and yielded 15% positive samples in the insecticide and 17.5% positives in the control samples.

Sporadically, *H. obsoletus* individuals were identified on the yellow sticky traps, the first catches originated from the sampling interval June 24 to July 1 (Online resource 11).

#### Rottersdorf 2020

**Fig. 7:**
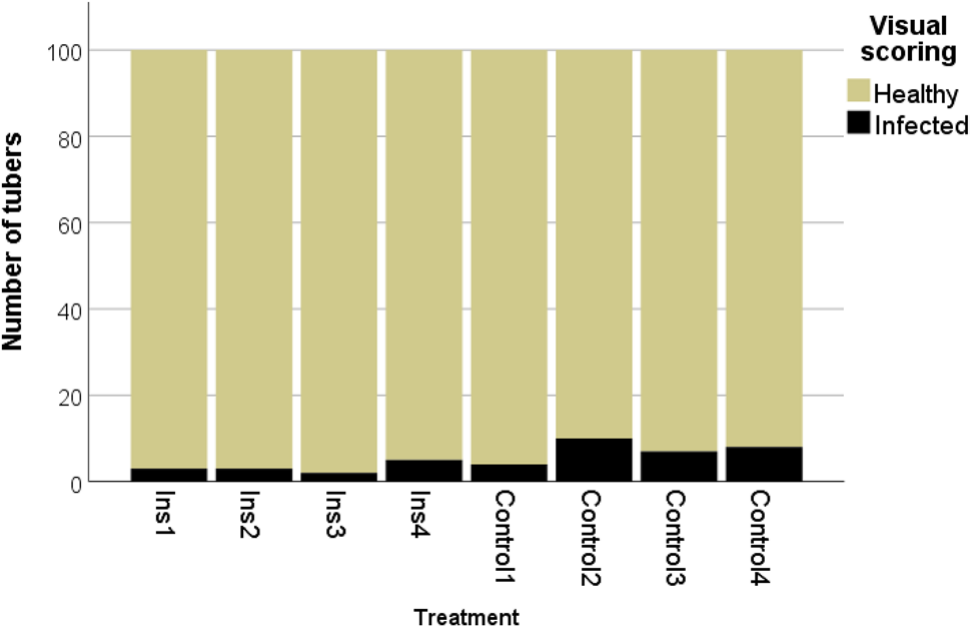
Rottersdorf 2020: Assessment of tubers for symptoms of ‘*Ca.* P. solani’. Applied compounds: Ins: Lambda-Cyhalothrin + Acetamiprid, Cypermethrin, Esfenvalerate

The field observations on July 30 revealed few symptomatic plants only. On August 14, rates of symptomatic plants in the insecticide treatment of 8.3% and 25.8%, in the control plots of 14.2% and 40.0% were determined (Online resource 12). Statistical analysis identified the treatment, the scoring date and the position of the plot within the experimental setup as factors significantly affecting the health status of the plants (Treatment: Wald *χ*^2^ = 5.73 *df* = 1, *p* = 0.018; scoring date: Wald *χ*^2^ = 54.69, *df* = 1, *p* = 0.000; position: Wald *χ*^2^ = 26.76. *df* = 1, *p* = 0.000; Table 7). Rates of symptomatic tubers were lower in the insecticide treatment, ranging from 3 to 5% in the insecticide treated plots and from 4 to 10% in the control plots (Fig. 7). The generalized linear model indicated a significant influence of the treatment on the rate of symptomatic tubers (Wald *χ*^2^ = 6.49, *df* = 1, *p* = 0.011; Table 7). PCR using a nested approach targeting the stamp gene confirmed these trends with 17.5% *Stamp* positive results in the control and 10% in the insecticide. Few *H. obsoletus* were captured on the yellow sticky traps, at maximum two individuals per trap were recorded from the sampling interval July 1-July 9 onwards (Online resource 13).

#### Maissau 2021

No visually discernible symptoms of stolbur were recorded in any of the examined plants during the visual inspections on July 30 and on August 19. In the whole field only five symptomatic plants were observed, all outside the examination area. Inspection of the tubers led to rates of symptomatic tubers of 2.0 and 4.0% for the insecticide treatment and 3.5 and 7.0% for the control, respectively (Online resource 14). The generalized linear model indicated a significant effect the factors treatment and position within the experimental design on tuber symptoms (Treatment Wald *χ*^2^ = 5.02. *df* = 1, *p* = 0.025; position: Wald *χ*^2^ = 7.39. *df* = 1, *p* = 0.007; Table 6). With nested Stamp PCR only a single positive tuber could be detected in control 2. Not a single *H. obsoletus* individual was present on any of the yellow sticky traps.

## Discussion

To the best of our knowledge, this is the first report on the efficacy of insecticides and repellents in preventing the spread of ‘*Ca*. P. solani’ including both laboratory and field experiments. The first objective of phytoplasma management by insecticides is to keep the vector population inside a field as low as possible and in that way to reduce pathogen transmission. In our laboratory study, all of the tested insecticides except spirotetramate significantly reduced insect survival as compared to the control. They might therefore more or less efficiently reduce insect survival and in consequence phytoplasma spread. Out of the tested compounds, especially the pyrethroids and the neonicotinoid acetamiprid caused high vector mortality, even when the spraying residues were already 4–5 days old.

A mere reduction of the insect populations inside the crop, however, is likely not enough for a sufficient pathogen restriction because the insect vectors constantly move into the potato fields from outside. In consequence, compounds protecting plants from becoming infected by disrupting the insect feeding behaviour in a shorter time than the minimum IAP are desirable. The IAP for *H. obsoletus* transmitting ‘*Ca*. P. solani’ is not well studied, in case of transmission to grapevine a minimum IAP of 3–6 h has been estimated (Bressan et al. 2007). A higher susceptibility of potato or vegetable species to the pathogen and, in consequence, a shorter IAP cannot be ruled out. It seems likely that the quicker the insecticide prevents vector feeding the greater the chance to reduce pathogen transmission. In our experiments, the compounds lambda cyhalothrin and deltamethrin led to an insect knock-down followed by mortality of 100% or close to 100% already after one hour of exposure. Within three hours, also acetamiprid in both concentrations, flupyradifuron and sulfoxaflor largely killed the planthoppers. Aged residues of acetamiprid, lamda-cyhalothrin and flupyradifuron still acted quickly. Data on insect mortality largely coincided with the phytoplasma transmission in these experiments. In tests including the above mentioned compounds and esfenvalerate all or almost all *C.roseus* remained healthy. In contrast, plants treated with less effective and/or slower acting compounds such as cyantraniliprole and spinosad became infected roughly to the same extent as the experimental control. Interestingly, the highest rate of infected test plants was recorded for spirotetramate, although, likely to the low number of tests, no statistically significant difference to the water control was observed (data on statistical analysis not shown). High rates of diseased plants also occurred in experiments with the putative feeding deterrent quassia. These observations raise the question if treatments inducing feeding deterrence via changes in taste and smell but without affecting insect survival lead to an adverse effect. Such treatments could prompt the insects to an enhanced search behaviour and finally increase phytoplasma transmission. Comparable effects of insecticides on phytoplasma transmission were reported for laboratory studies on the leafhopper *Macrosteles quadripunctulatus* vectoring chrysanthemum yellows phytoplasma (CYP)*.* In transmission experiments including imidacloprid and organophosphates potted *Chrysanthemum carinatum* was significantly protected from infection (Saracco et al. 2008). All in all our laboratory study, together with literature reports, proves that insecticide applications might influence phytoplasma transmission, provided that the active agents (and their spray deposits) are (still) effective and fast acting.

Due to the difficulties in collecting a sufficient number of insects in the field, the laboratory study comprised one test procedure only. Admittedly, this approach had deficiencies in considering the different modes of action of the test agents. The used scheme, namely the insect release on the treated test plants two to four hours after application of the test products, was likely most suitable for identifying the effects of compounds with a rapid contact action. This was e.g. the case for the pyrethroids, which, upon contact, result in a rapid knock down and death of the target insects (Lybrand et al. 2020). The pyrethroid lambda–cyhalotrin was applied as encapsulated formulation Karate Zeon with enough stability to show knock down and high mortality still after 5 days. Acetamiprid, flupyradifurone and sulfloxaflor are compounds with contact effect and systemic translocation in the plant (Yamada et al. 1999; Nauen et al. 2015; Bacci et al. 2018). Accordingly, all these agents were effective in the setup of the current study. The procedure also revealed an effect of flonicamid on *H. obsoletus* survival and pathogen transmission. This compound is particularly active against hemipterous pests and inhibits the insects’ feeding behaviour within a short time (Morita et al. 2007). Likely, the test scheme was also adequate for evaluating the effects of the included organic insecticides. At least some direct contact effect through the body surface has been reported for spinosad as well as for *Quassia amara* extract (Grdiša and Gršić 2013; Bacci et al. 2016), albeit these agents were not very effective against *H. obsoletus* in the actual study. Azadirachtin is considered as pronounced antifeedant provoking behavioural avoidance in many insect species (Kilani-Morakchi et al. 2021), a fact that might have resulted in the relatively low rate of infected *C. roseus* in the current experiments.

In contrast, out test set up was less suitable to identify effects of active compounds with predominantly systemic activity. This was particularly the case for spirotetramat, which becomes effective after absorption and translocation in the plant, a process requiring more than several hours to days. Moreover, it particularly affects juvenile stages and adult fecundity (Brück et al. 2009). However, in the current study, the insects were released hours after application of the test compounds, the complete observation period lasted five days only, and due to the subterraneous lifestyle of the *H. obsoletus* instars, only adults were included. Likely, these factors contributed to the low effect of this agent in the current tests. The compound chlorantraniliprole mainly acts by ingestion and is to some extent transported in the plant (Lahm et al. 2009). In the current study, the effect on *H. obsoletus* and pathogen transmission was relatively low, likely, also in this case, due to a lack of time for systemic movement of the compound within the test plants.

Some reducing effect on the rate of infected *C. roseus* as compared to the control was also observed for the particle film forming compounds kaolin and diatomaceous earth. For a long time it has been known that kaolin particle film coating results in a hostile environment for insects and in consequence reduce insect movement, feeding and egg-laying (Glenn and Puterka 2005). Diatomaceous earth is based on fossilised unicellular algae and contains amorphous silicon dioxide. The porous particles have an abrasive effect and a high capacity for absorption of lipids. Contact damages the insects ‘wax coating’ and leads to desiccation. In addition, the compound can interfere with the insects’ settling, probing and feeding behaviour (Korunic 1998).

Simultaneously with and subsequently to the laboratory experiments, we made efforts to transfer the achieved laboratory results to field conditions. In 2019, the insecticide applications comprised the compounds lambda –cyhalotrin and esfenvalerate. We included pyrethroids only, despite the fact that in practice active agents from different IRAC (Insecticide Resistance Action Committee) groups ought to be selected. However, at the start of the field experiments, the laboratory results outlined above were only partly available and we regarded pyrethroids due to their knock down effect as particularly promising. Other compounds, such as chlorpyriphos, were ruled out because of their expiring registration. Mineral oil and diatomaceous earth, were included in the study because of their lower environmental impact. Both in Maissau and in Rottersdorf in 2019, at the beginning of the experiments during the visual assessments in July, the insecticide effect seemed promising. The treated plots looked healthier and the insecticide treatment reduced the rates of infected plants roughly by half. Until the middle of August, however, in Rottersdorf the vast majority of plants in all plots had collapsed. In Maissau, at the time, the rate of infected plants was almost halved in comparison to the experimental control, but the rate of diseased plants still reached 35%. In Rottersdorf, the high share of symptomatic plants in the insecticide treatment was also reflected in the status of the tubers. No or only marginal differences to the experimental control were evident, neither in respect to gummy tubers nor in respect to PCR results. In Maissau, statistical analysis proved a significant insecticide effect on the health status of the tubers, but from a practical point of view, the rate of gummy tubers (on average 17%) was too high also in this location. Rates of PCR-positive tubers were even slightly higher (22.5%). The particle films formed by diatomaceous earth had no or almost no effect from the first visual scoring onwards. Similar results were obtained for the mineral oil treatments.

In 2020, based on the experiments in 2019 and on the registration situation, treatments included the insecticide compounds lambda-cyhalothrin, acetamiprid and cypermethrin. In this year, however, all over Lower Austria disease incidence and severity sharply declined in comparison to the years before (data not shown). Also in our two experiments, insect populations were smaller and the flight period started later than in 2019. In Maissau, disease incidence in the field and rates of symptomatic tubers were low and marginally influenced by the treatments. In Rottersdorf, despite or maybe because of the lower infection pressure a treatment effect was clearly visible. Both, the incidence of field symptoms and the share of gummy tubers were roughly halved.

In Maissau in 2021, the infection pressure was even lower than in 2020. Not a single vector individual stuck on the yellow traps and no symptomatic plants occurred in the rows previously defined for visual scoring. Sporadically, single infected plants were observed in other parts of the field only. Nevertheless, a certain treatment effect on the rates of gummy tubers was visible and confirmed by PCR analysis.

Over all experiments, a good agreement between captures of *H. obsoletus* and disease incidences indicated that this insect species was the most relevant pathogen vector. In 2019, constant insect captures on the yellow sticky traps resulted in high disease rates, whereas in 2021, an obviously low insect population (without captures on yellow sticky traps) was related to low numbers of infected plants. In single cases, other potential vectors, namely *Reptalus* panzeri, *R. quinquecostatus* and *Pentastiridius leporinus* were also present on the yellow sticky traps (data not shown), however, due to their rare occurrence, their impact on disease spread in the test fields was likely negligible.

Overall, the results of the current study demonstrate that the insecticide treatments influenced phytoplasma transmission also in field conditions. Maintaining plant health in the course of the vegetation period, however, proved challenging. Putatively, hot temperatures and strong solar radiation in July reduced the insecticide efficacy in a shorter time than the interval of eight days between the treatments. In consequence, the aged spray residues provided no full protection against the pathogen transmission. During the long insect flight period with a constant infection risk the share of diseased plants grew steadily. Moreover, pyrethroids, the predominantly used insecticides in the current study have a contact effect only. In case of spraying densely growing potatoes, a complete coverage with the spray liquid is difficult to achieve, particularly on the stems and the midribs on the underside of the leaf. The vector insects, however, prefer these parts of the plant for feeding. In consequence, an incomplete coverage of the plants with the insecticide might have contributed to the infection rates. Divergent results of the experiments in Maissau and Rottersdorf in 2019 are likely attributable to the two different potato varieties. In Rottersdorf, the planted variety was ‘Tosca’, which proved as extremely susceptible in the course of this stolbur outbreak. Eurostarch, the variety in Maissau, turned out as moderately susceptible (Brader, unpublished).

Comparing the starting times of the treatments with the insect captures in the field in 2019, it emerged, that in all experiments the insecticide treatment had started around 7–10 days earlier than relevant insect populations occurred in the field. The reason for the time lag between the start of the flight period at the roadsides analysed for determination of insect development and insect presence in the potato fields remained unclear. Likely, in that year, the first insecticide applications could have been omitted without a relevant reduction in efficacy.

Treatments with mineral oil and diatomaceous earth were largely ineffective. Already in the cage experiments, despite a full coverage of the plants, the effect of the particle films was relatively low. Likely, in the field the effect of diatomaceous earth was additionally impaired by the fact that a dense coverage of the entire plants with the spray suspension was not achieved using the spraying equipment available at the two commercial farms. Although mineral oils are known to obstruct the insects’ tracheal structures and provoke structural changes in the cuticle and residual oil films discourage insects from feeding (Buteler and Stadler 2011), these effects were not sufficient to prevent the transmission of ‘*Ca*. P. solani’ in the field. Admittedly, with respect to statistical processing of the data, in all our experiments higher numbers of repetitions would have been desirable. However, as already outlined, the trials were carried out in commercial farms, where higher numbers of plots were not feasible due to temporal and technical restrictions.

From a practical point of view, our experiments showed that treatments with the insecticides alone are certainly not sufficient to manage potato stolbur in years with high disease pressure and in case of susceptible varieties. A little more encouraging results might be expected in situations with lower disease pressure and less susceptible varieties. In the last two or three years in Austria, we witnessed a rapid decline of the bindweed associated *H. obsoletus* populations and correspondingly, an endemic disease phase. Therefore, we were unable to clarify the question whether application of insecticides could and should be one component of a future management programme together with other measures, particularly the planting of less susceptible varieties. The results of this study and further field observations during the epidemic disease phase in Austria indicate a great variability of potato varieties in respect to their stolbur tolerance (data not shown).

In any case, the rather dense spraying intervals and the considerable amount of plant protection agents used in the current study likely entailed a broad range of side effects on the environment, while a substantial or even complete disease suppression was not achieved. Further studies must clarify if insecticide treatment, for example a reduced number of applications right before or at the flying peak of *H. obsoletus,* could be a justifiable measure leading to a proportionate disease reduction.

## Supplementary Information

Below is the link to the electronic supplementary material.

Supplementary file 1 (PDF 2144 kb)
