# Supplementary material for: Effects of insecticides and repellents on the spread of ‘Candidatus Phytoplasma solani’ under laboratory and field conditions
Source: J Plant Dis Prot (2006). 2023 Jul 4;130(5):1057–74. doi: 10.1007/s41348-023-00768-y (PMC10421767; doi:10.1007/s41348-023-00768-y)
Supplement: Supplementary file 1 — (PDF 2144 kb) [file 41348_2023_768_MOESM1_ESM.pdf]

Effects of insecticides and repellents on the spread of ‚*Candidatus* Phytoplasma solani‘ under laboratory and field conditions

Monika Riedle-Bauer[1], Günter Brader[2*]

[1]Federal College and Research Institute for Viticulture and Pomology Klosterneuburg, Wienerstraße 74, 3400 Klosterneuburg, Austria; Monika.Riedle-Bauer@weinobst.at

[2]Austrian Institute of Technology, Konrad-Lorenz-Straße 24, 3430 Tulln, Austria

[*] Corresponding author: guenter.brader@ait.ac.at; phone: +43(0) 50550-4669; fax: +43(0) 50550-3666

Abstract

Recent outbreaks of '*Candidatus* Phytoplasma solani' resulted in severe losses in potatoes, vegetable crops and grapevines in certain regions of Austria and constituted a major challenge for seed potato production. Therefore, the effects of various insecticides and insect deterrents on pathogen spread were studied both in laboratory and field experiments over the period from 2018 to 2021. In laboratory transmission experiments field captured *Hyalesthes obsoletus* were caged on differently treated *Catharanthus roseus* for five days. The insecticdes lambda-cyhalothrin, deltamethrin, esfenvalerate, acetamiprid and chlorpyriphos showed the most rapid impact on insect survival and fully prevented phytoplasma transmission. The particle film forming products kaolin and diatomaceous earth had some effect. A transfer of the promising laboratory results to potato fields, however, was achieved to a limited extent only. Treatments with pyrethroids and acetamiprid every 8-10 days over the flight period of *H. obsoletus* roughly halved the number of symptomatic plants and tubers in case of moderately susceptible varieties and moderate infection pressure. In the event of susceptible varieties and high disease pressure, treatment effects were hardy discernible. In practical terms the experiments indicate, that insecticide applications alone are not sufficient to mitigate the disease. Spraying of diatomaceous earth and mineral oil had no effect on disease incidence in the field.

Online resource 1: Origin and qPCR-infection rate of *Hyalesthes obsoletus* specimen included in the laboratory experiments

Sampling location	Result of qPCR analysis<br>N° positive *H.obsoletus* /N° tested *H. obsoletus* /% positive specimen
Naglern 1, roadside along infected potato fields: 48.491982, 16.369135	12/24/50.0%
Naglern 2; roadside along infected potato fields: 48.488895, 16.369630	10/30/33.0%
Weinsteig; roadside: 48.458317, 16.393174	9/19/47.4%
Wolkersdorf, vineyard: 48.391074, 16.5260004	4/8/50.0%

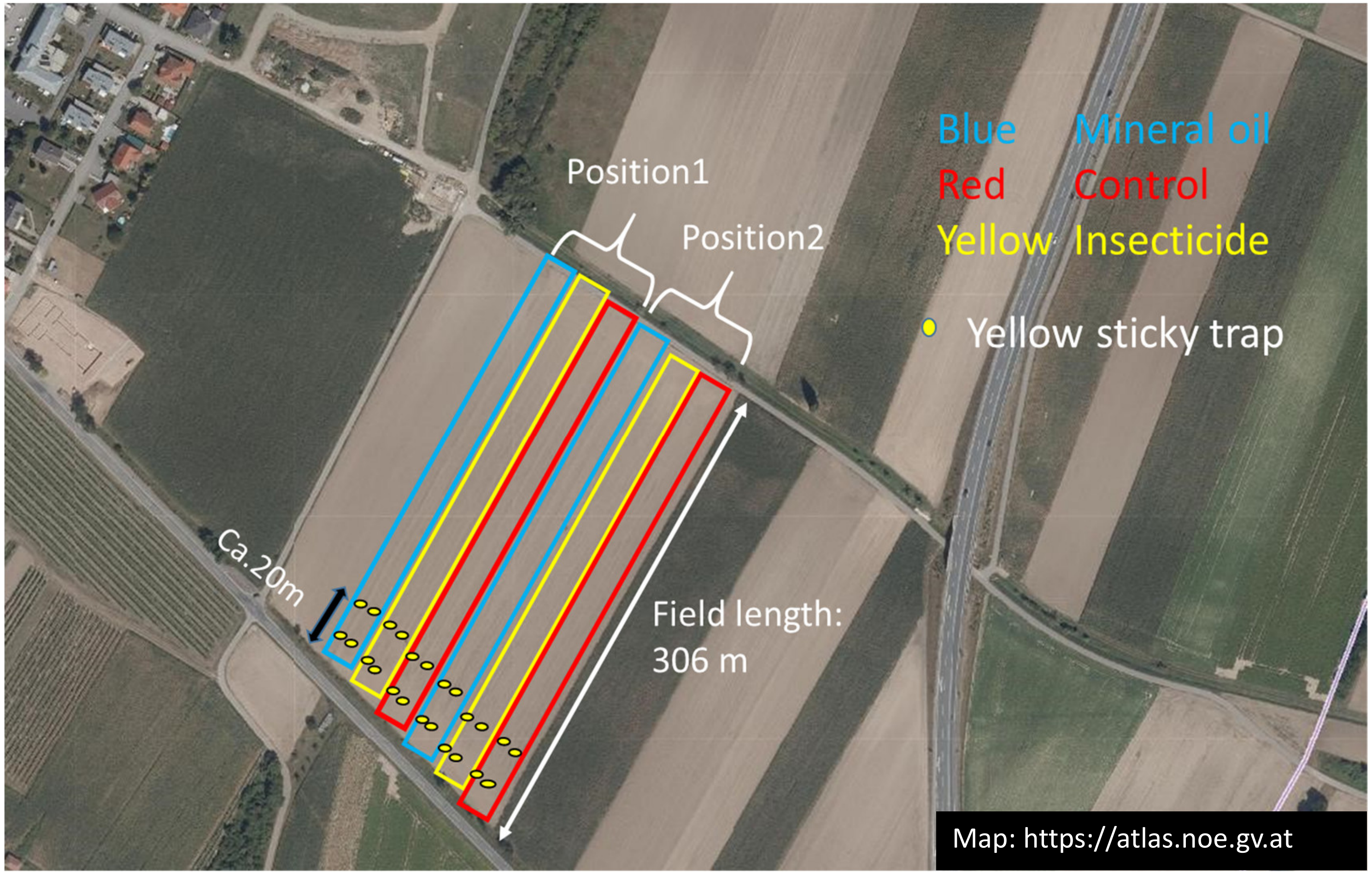

Online resource 2: Layout of experimental field in Maissau 2019

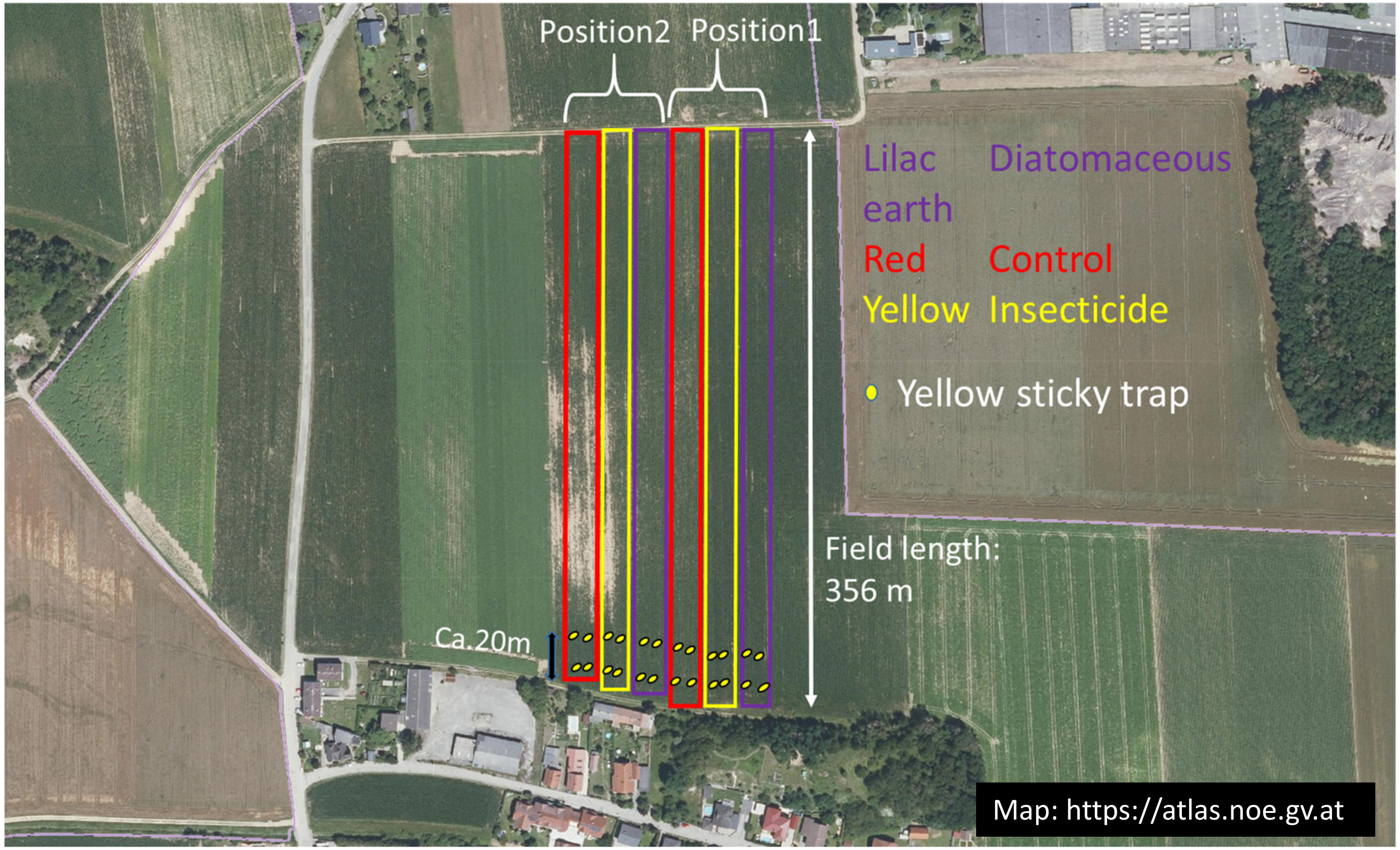

Online resource 3: Layout of experimental field in Rottersdorf 2019

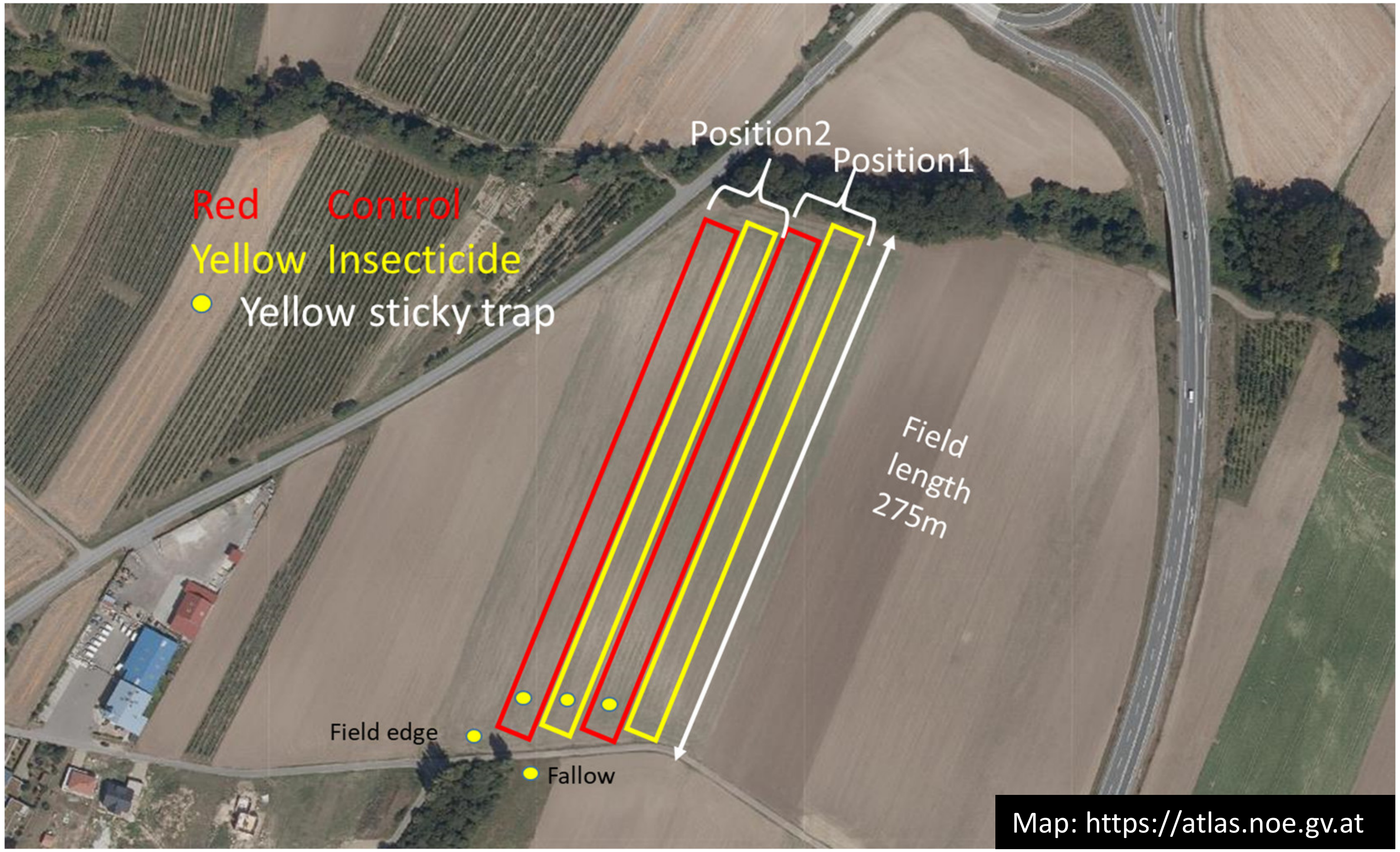

Online resource 4: Layout of experimental field in Maissau 2020

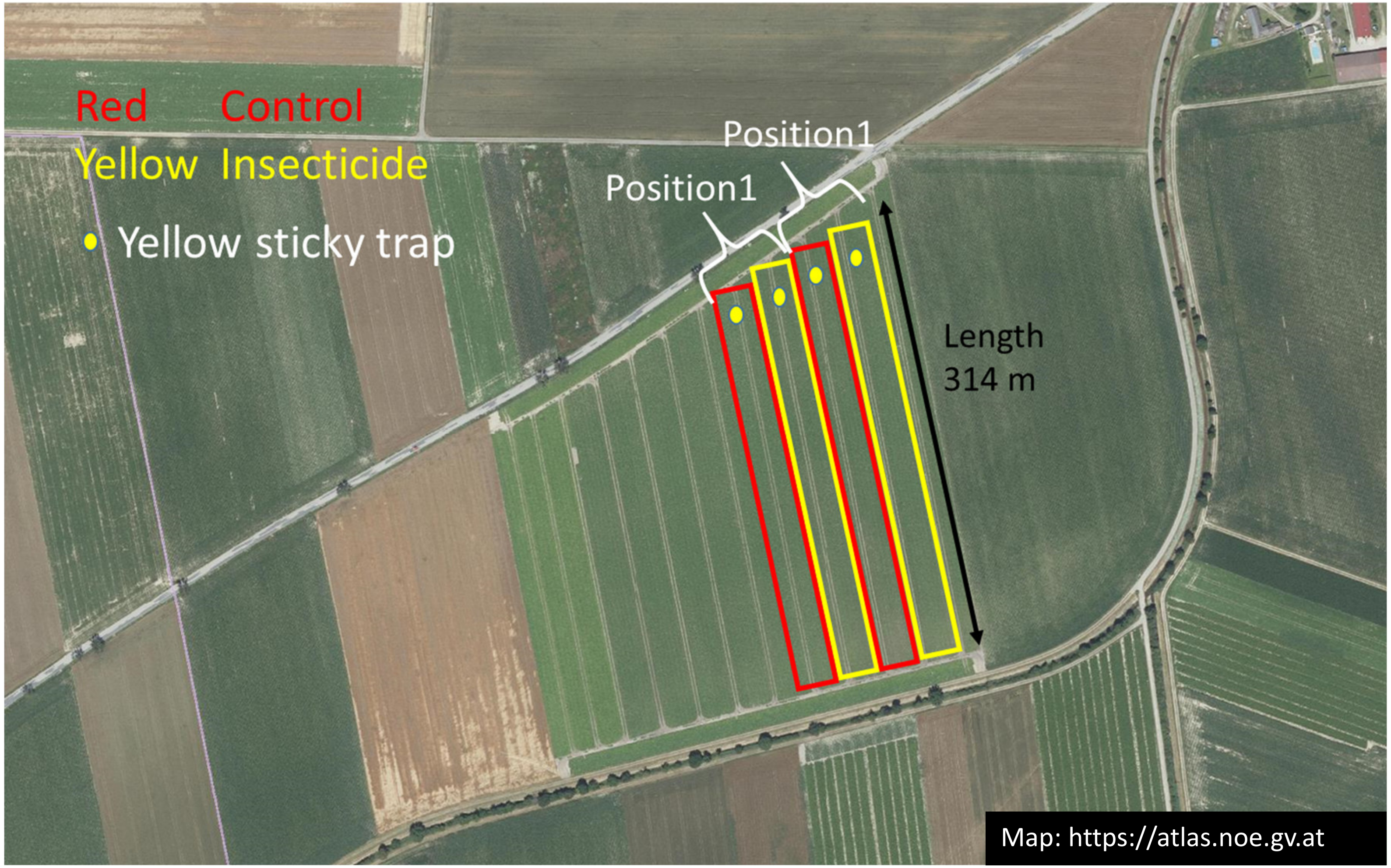

Online resource 5: Layout of experimental field in Rottersdorf 2020

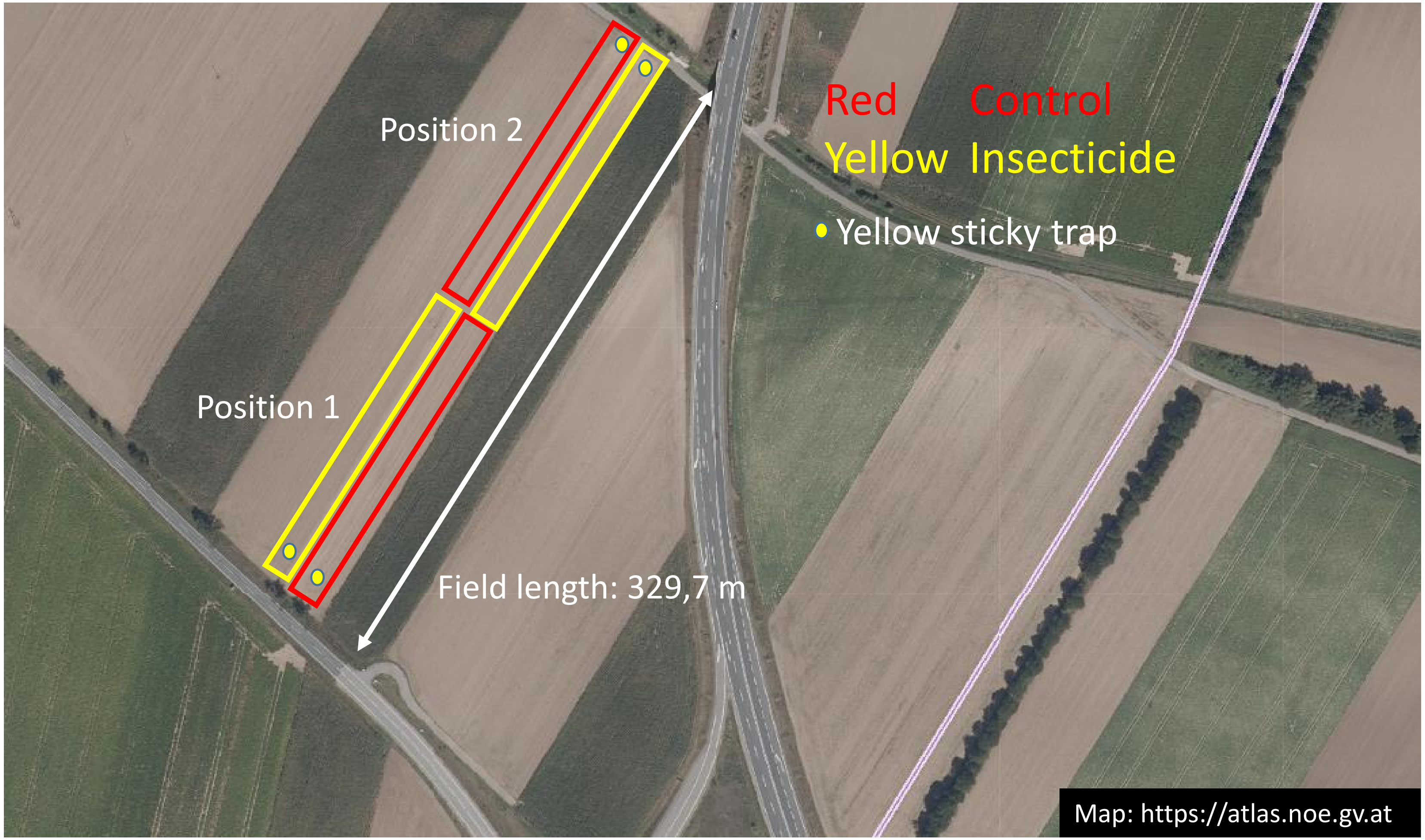

Online resource 6: Layout of experimental field in Maissau 2021

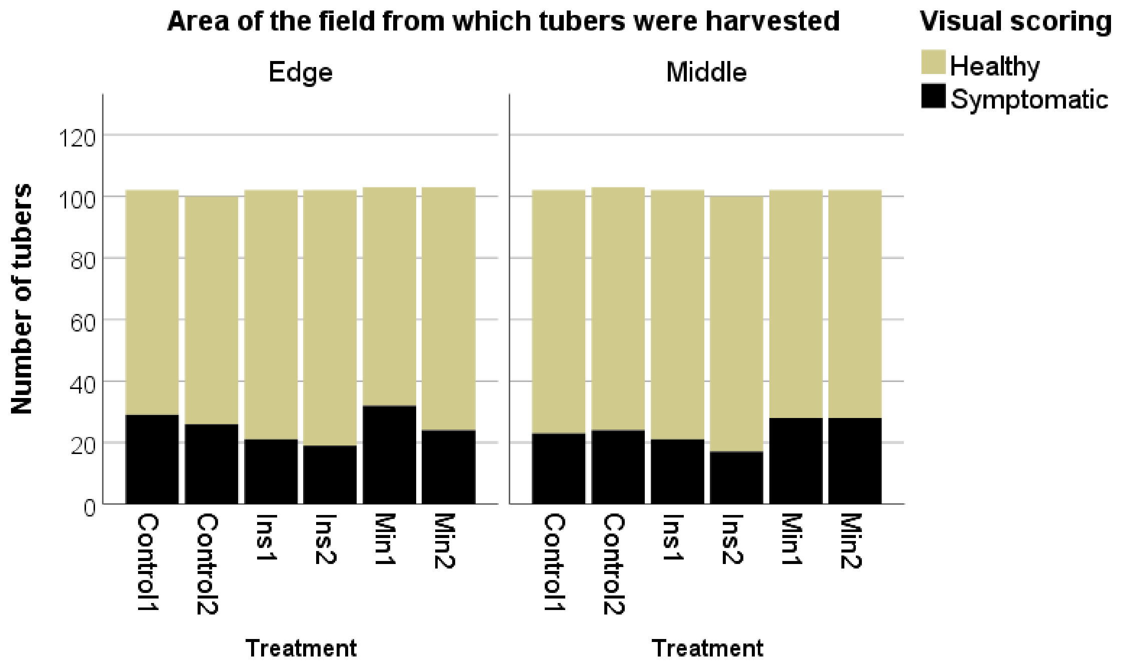

Online resource 7: Maissau 2019: Assessment of tubers collected at the edge and in the middle of each plot for symptoms of '*Ca.* P. solani'. Applied compounds: All treatments: Acetamiprid against *L. decemlineata*; Ins: Esfenvalerate, Lambda-Cyhalothrin, Min: White mineral oil.

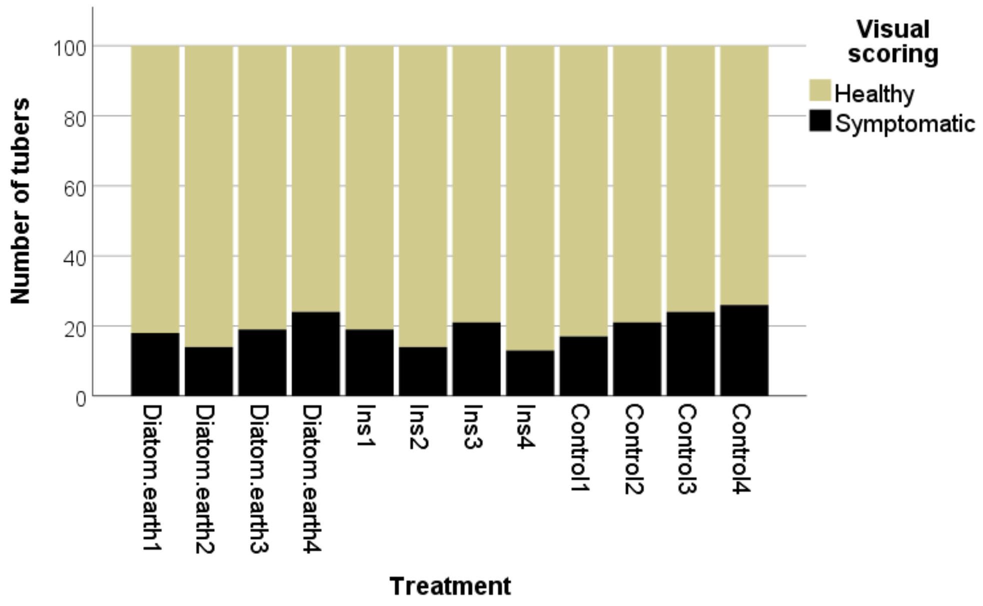

Online resource 8: Rottersdorf 2019: Assessment of tubers for symptoms of '*Ca.* P. solani'. Applied compounds: Ins: Esfenvalerate, Lambda-Cyhalothrin; Diatom.earth: Diatomaceous earth.

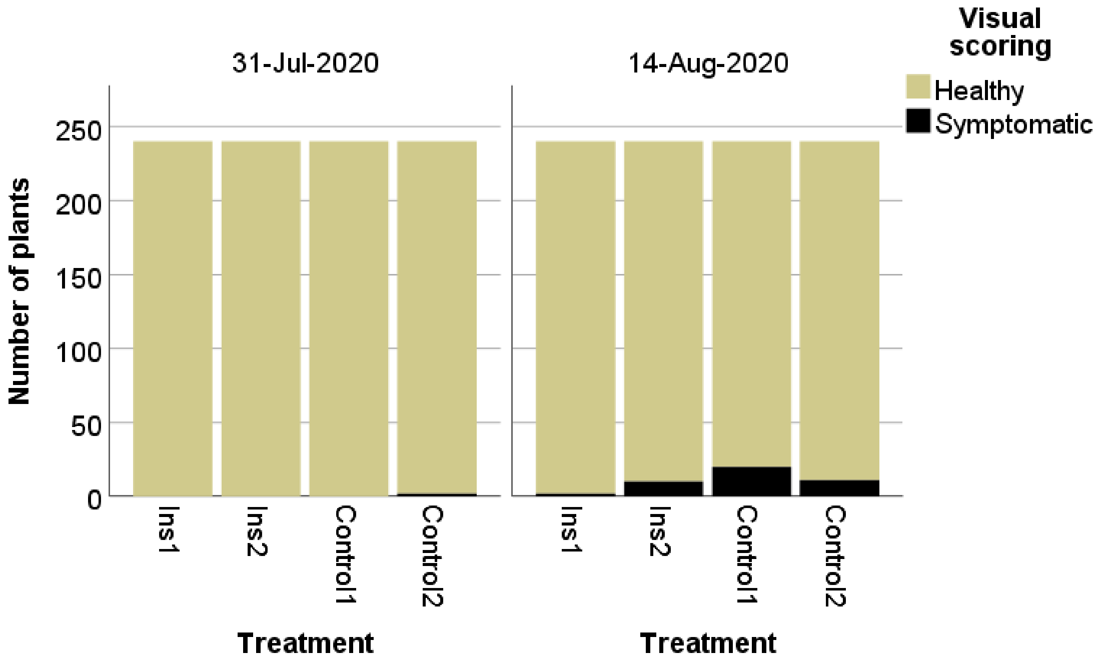

Online resource 9: Maissau 2020: Rates of visually diseased plants in the field in the course of the experiment. Applied compounds: Ins: Lambda-Cyhalothrin+Acetamiprid, Cypermethrin, Esfenvalerate.

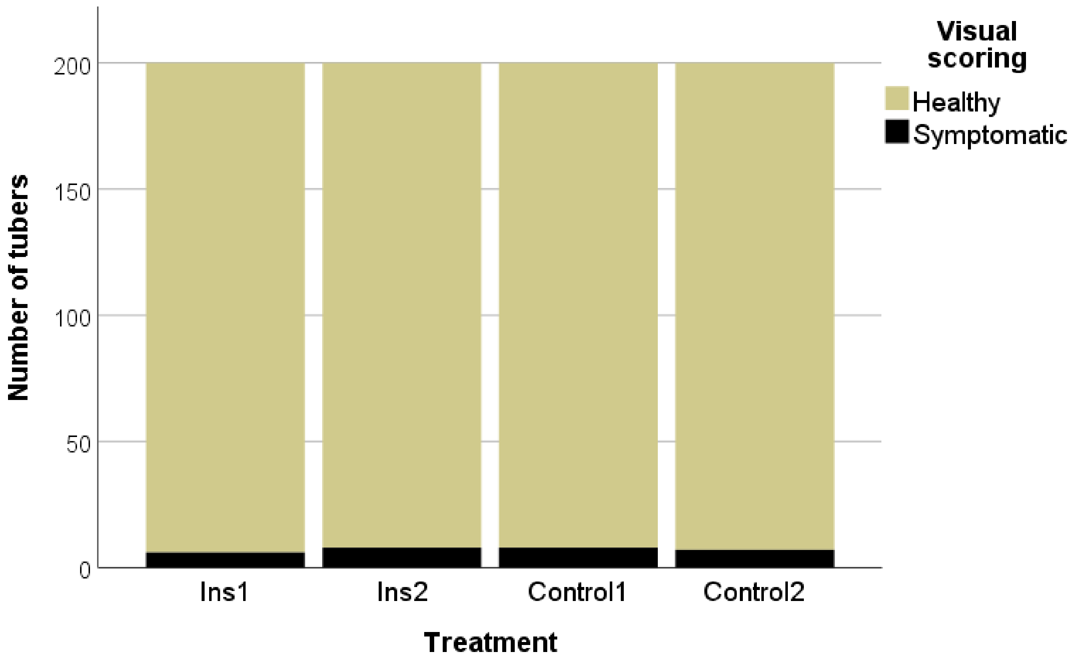

Online resource 10: Maissau 2020: Rates of symptomatic tubers. Applied compounds: Ins: Lambda-Cyhalothrin+Acetamiprid, Cypermethrin, Esfenvalerate.

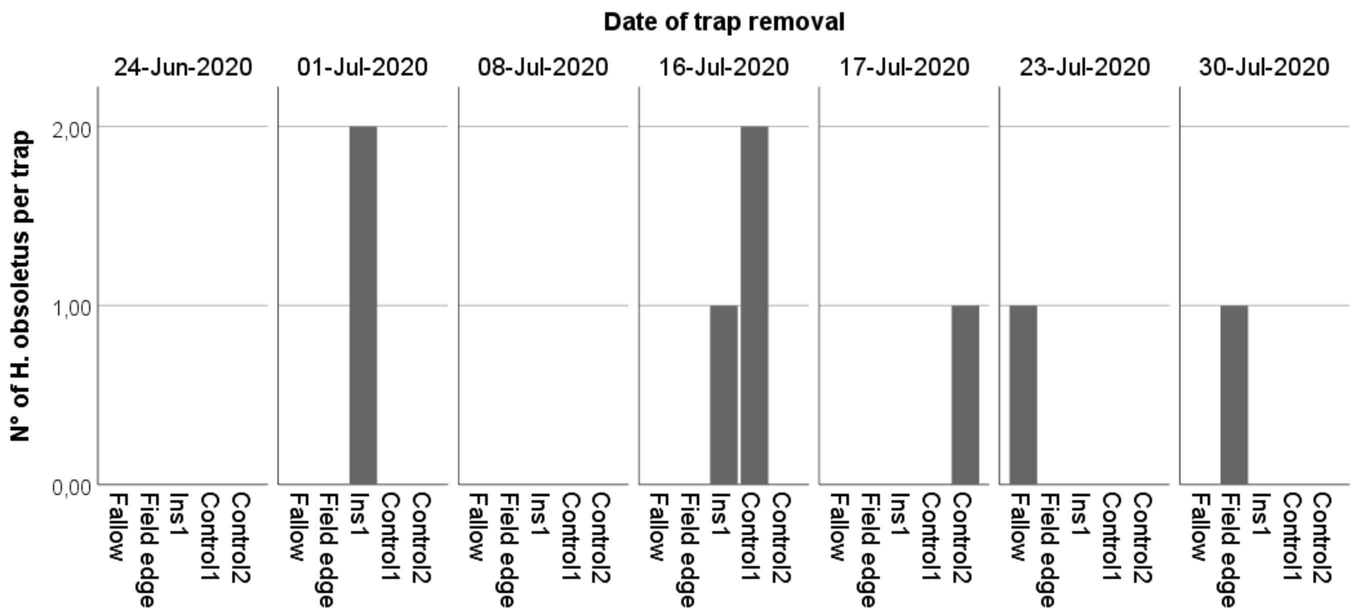

Online resource 11: Maissau 2020: Insect captures on yellow sticky traps. Applied compounds: Ins: Lambda-Cyhalothrin+Acetamiprid, Cypermethrin, Esfenvalerate.

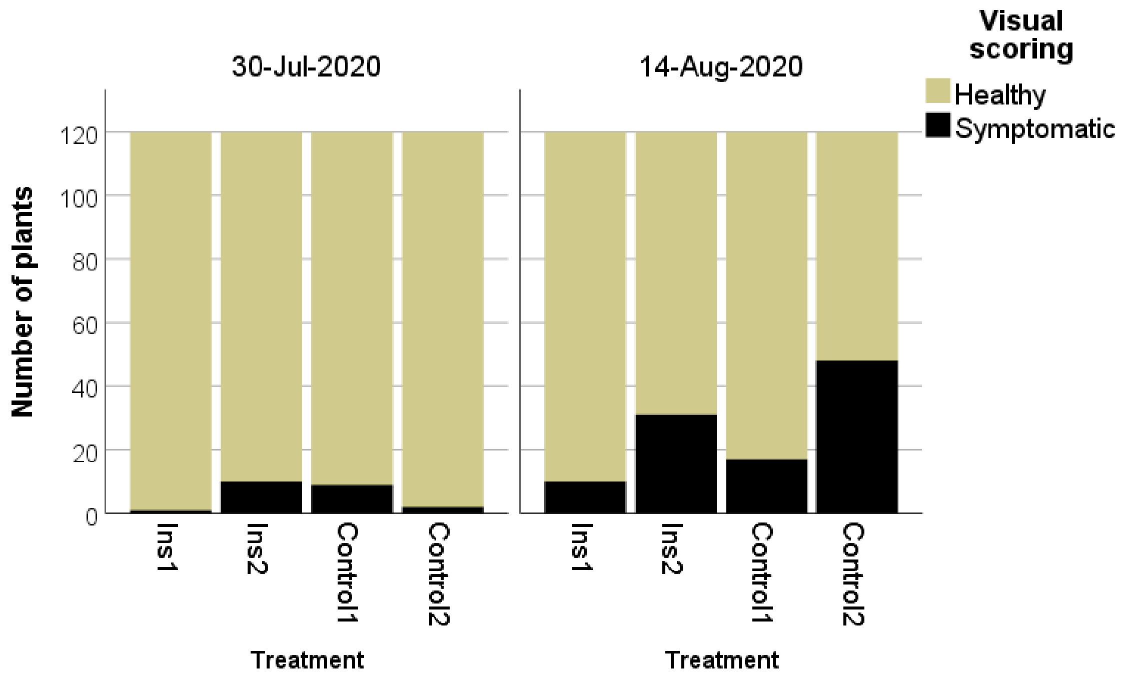

Online resource 12: Rottersdorf 2020: Rates of visually diseased plants in the field in the course of the experiment. Applied compounds: Ins: Lambda-Cyhalothrin+Acetamiprid, Cypermethrin, Esfenvalerate.

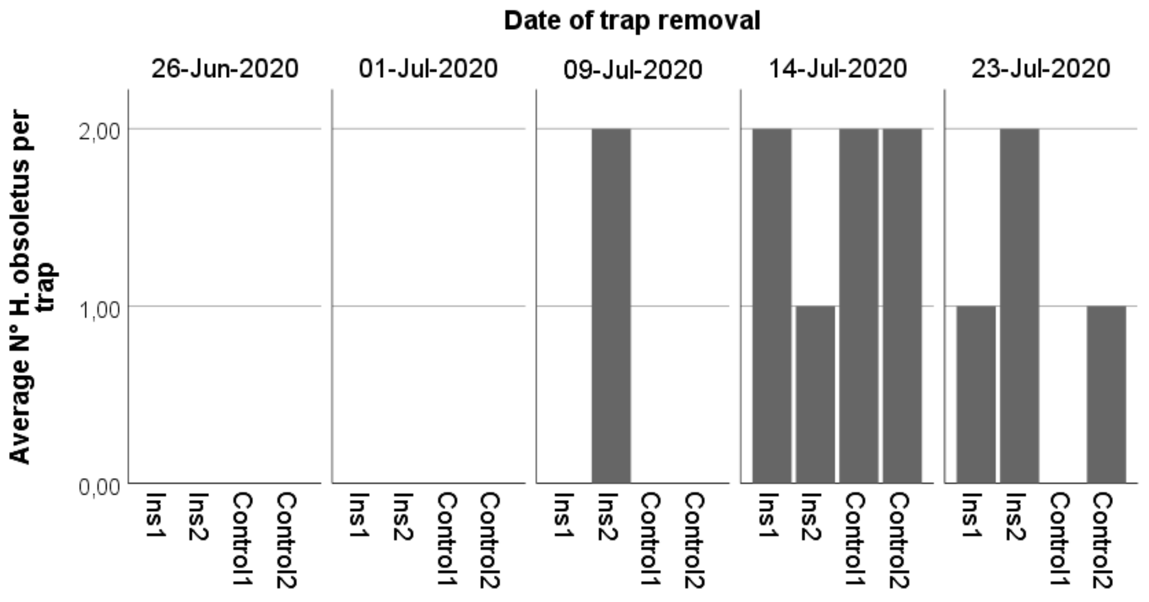

Online resource 13: Insect captures in Rottersdorf 2020. Applied compounds: Ins: Lambda-Cyhalothrin+Acetamiprid, Cypermethrin, Esfenvalerate.

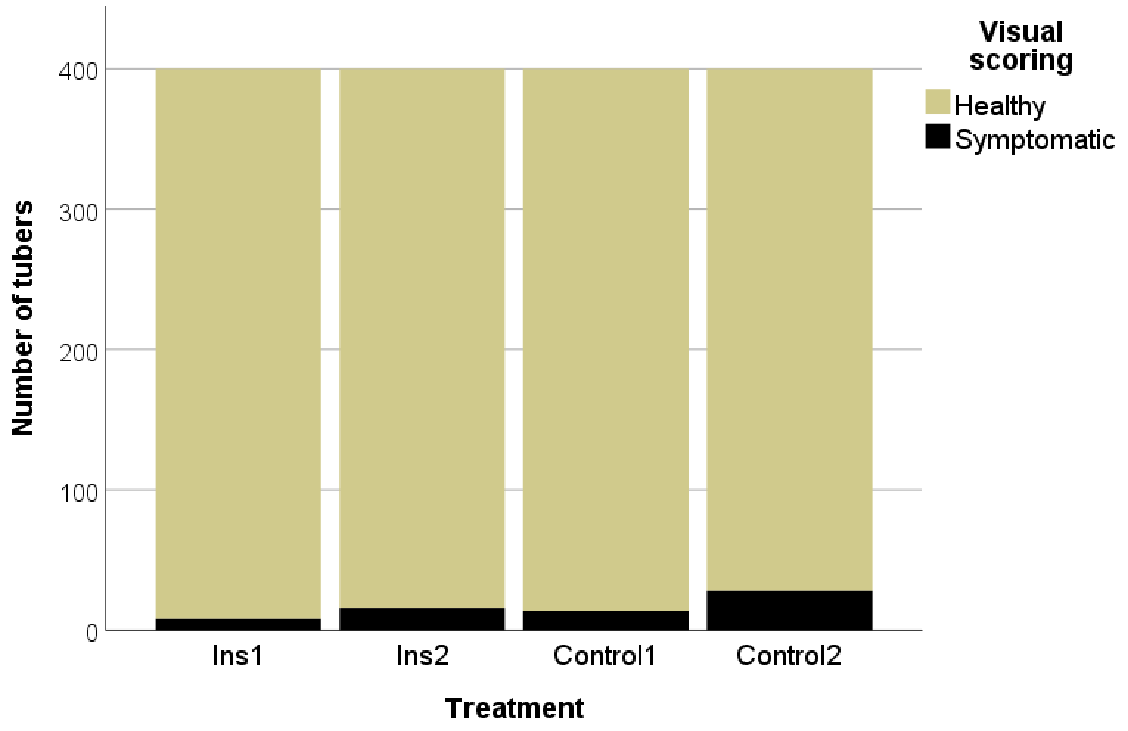

Online resource 14: Maissau 2021: Assessment of tubers for symptoms of '*Ca.* P. solani'.Applied compounds: Chlorantraniliprole against *L. decemlineata*, Esfenvalerate+Acetamiprid, Lambda-Cyhalothrin.
